# Intrinsic and extrinsic noise of gene expression in lineage trees

**DOI:** 10.1038/s41598-018-35927-x

**Published:** 2019-01-24

**Authors:** Philipp Thomas

**Affiliations:** Department of Mathematics, Imperial College London, London, SW7 2AZ UK

## Abstract

Cell-to-cell heterogeneity is driven by stochasticity in intracellular reactions and the population dynamics. While these sources are usually studied separately, we develop an agent-based framework that accounts for both factors while tracking every single cell of a growing population. Apart from the common intrinsic variability, the framework also predicts extrinsic noise without the need to introduce fluctuating rate constants. Instead, extrinsic fluctuations are explained by cell cycle fluctuations and differences in cell age. We provide explicit formulas to quantify mean molecule numbers, intrinsic and extrinsic noise statistics in two-colour experiments. We find that these statistics differ significantly depending on the experimental setup used to observe the cells. We illustrate this fact using (i) averages over an isolated cell lineage tracked over many generations as observed in the mother machine, (ii) population snapshots with known cell ages as recorded in time-lapse microscopy, and (iii) snapshots with unknown cell ages as measured from static images or flow cytometry. Applying the method to models of stochastic gene expression and feedback regulation elucidates that isolated lineages, as compared to snapshot data, can significantly overestimate the mean number of molecules, overestimate extrinsic noise but underestimate intrinsic noise and have qualitatively different sensitivities to cell cycle fluctuations.

## Introduction

Cellular behaviour varies substantially from cell to cell and over time^1–3^. Identifying the sources of these fluctuations can help reveal the function of gene circuits and signalling networks and understand how clonal cells diversify their responses. When quantifying these fluctuations in gene regulatory networks, it is often convenient to break down the experimentally observed variability into functional components. Commonly one wishes to separate fluctuations inherent in the circuit dynamics itself, called *intrinsic noise*, from fluctuations that arise from embedding the circuit in the environment of the cell, called *extrinsic noise*.

A possible resolution to this problem is to place and simultaneously measure a second independent copy of the circuit in the cell, as has been realised in bacteria^1^, yeast^4^, mammalian^5^ and plant cells^6^. The difference between the two circuit copies reflect the intrinsic noise whereas their correlations measure the extrinsic noise component. Intrinsic noise arises from the random nature of the involved biochemical reactions. Extrinsic noise originates from factors affecting both circuits in the same way. These can, for instance, be modelled by reaction rates that fluctuate between cells or over time due to shared resources, promoter architecture or upstream pathways. Such sources of extrinsic noise have been studied extensively in the literature^7–16^.

A less commonly studied but equally important source of extrinsic noise is the population dynamics^17^. Since intracellular molecule numbers must double over the cell division cycle, a two-fold variation of expression levels is expected from cell proliferation alone. Moreover, the cell cycle itself is subject to tremendous variation providing an additional source of extrinsic variability. For example, generation times in *Escherichia coli*^18^, budding yeast^19^ and mammalian cells^20^ vary about 30–50%. These sources should therefore prevail in growing cells, populations and tissues.

Modelling approaches for understanding the cell cycle effects on gene expression noise are only recently being developed^21–27^. These studies are often restricted to a single isolated cell observed over successive cell divisions and measuring variability over time, similar to a lineage in the mother machine^28^. Many experiments, however, report cell-to-cell variability across snapshots of an exponentially growing cell population. These approaches either use time-lapse microscopy^29,30^ or analyse snapshots with distributed cell cycle stages as in flow cytometry, smFISH or similar techniques^31–33^.

Recent studies elucidated that population snapshots and lineages can significantly deviate in their statistics^27,34^. To-date, however, there exists no general analytical framework with which to quantify the gene expression fluctuations in populations. We are thus lacking the means with which to understand, compare and integrate measurements from different experiments such as mother machines, time-lapse microscopy or fixed-cell images. Agent-based approaches allowing to track the expression levels of each individual cell in a growing population are ideally suited to address this issue.

In this manuscript, we develop such an approach to analyse stochastic biochemical reaction networks in a growing and dividing cell population. In this framework, an agent is represented by a cell whose biochemical composition changes due to stochastic reaction kinetics and cell divisions. A distinguishing feature of this theory is that it takes into account the cell age – the time since the last division – which is accessible from time-lapse microscopy or cell cycle markers. We show how to analytically characterise the joint distribution of cell age and molecule content per cell in a lineage tree. We then derive the exact moment equations of this model.

Since stochastic models are rarely solvable, we propose an analytically tractable approximation to mean and covariances. Intrinsic and extrinsic noise sources as they are measured using two-reporter systems are in-built in the agent-based approach, and we explain how to decompose the apparent noise into the respective components. We further elaborate on the decomposition in cases where the cell age is unknown, a situation commonly encountered when analysing data from population snapshots or flow cytometry. We demonstrate how to practically compute the noise decomposition, illustrate the results using a simple two-reporter system, and study how circuit dynamics can be tuned to suppress either intrinsic or extrinsic fluctuations.

## Methods

**Figure 1:**
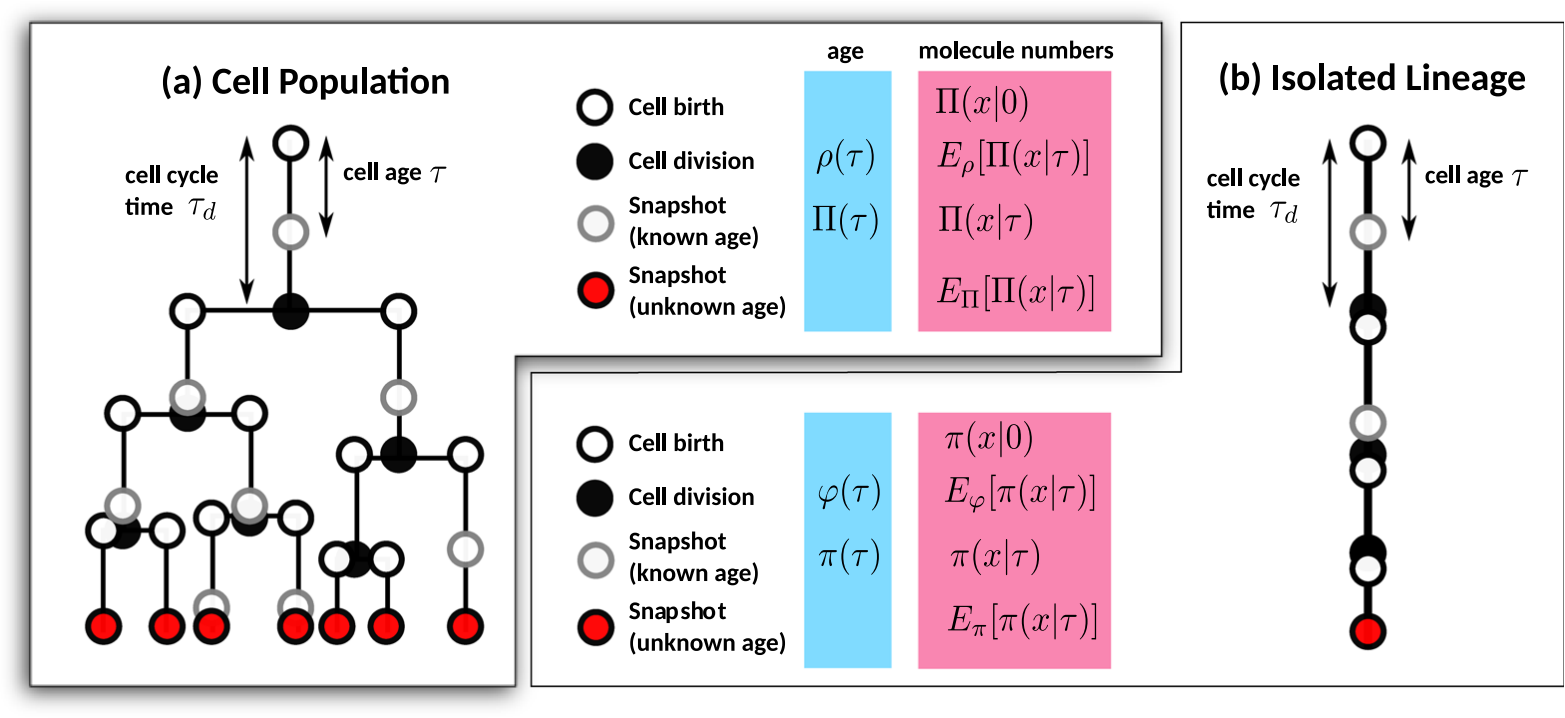
Single-cell statistics of cell populations and isolated lineages. (**a**) Distributions of the molecule numbers *x* measured in a cell of age *τ* across a lineage tree from time-lapse movies. The population dynamics is described by the age distribution Π(*τ*) and the distribution of interdivision times *ρ*(*τ*). Gene expression dynamics is described by the distribution Π(*x*|*τ*) for cells of age *τ* (open grey circles). Accordingly, the distributions are Π(*x*|0) at cell birth (open black circles) and *E*_*ρ*_[Π(*x*|*τ*_*d*_)] at cell division (filled black circles). The final state of the population (red dots) describes a snapshot with distributed cell ages *E*_Π_[Π(*x*|*τ*)] as obtained from static images or flow cytometry. (**b**) The corresponding statistics for a lineage following an isolated cell over time as observed in the mother machine.

There exist a variety of ways of how to pool data from different cells in lineage trees. In Fig. 1a, we summarise the different distributions that characterise tree-structured data. Time-lapse data resolves the age of each cell and hence enables measuring the statistics of interdivision times and molecule numbers at cell birth (open black circles), division (filled black circles) or at a specified position in the cell cycle (open grey circles). If the cell age is unknown, as for static images or flow cytometry data, only a snapshot of the present population is available (red dots). The corresponding measures for an isolated lineage of a single cell tracked over time, as in the mother machine, are summarised in Fig. 1b.

**Figure 2:**
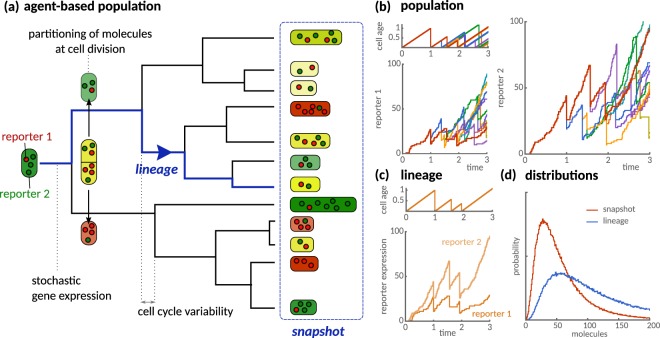
Agent-based model of clonal population dynamics with stochastic gene expression and cell cycle variability. (**a**) Illustration of a growing population as a branching process with stochastic interdivision times. Each cell expresses two identical but non-interacting reporters (green and red) that are partitioned randomly at cell division. Red and green cells express more molecules of either reporter, which indicates intrinsic variability between cells. Yellow cells express similar levels of reporter molecules, but their levels vary over the cell cycle resulting in extrinsic variability. A snapshot of the population (blue dashed box) quantifies the cell-to-cell variability across the population. A lineage (blue path) quantifies variability over time and tracks an isolated cell over successive cell divisions by randomly selecting one of the daughter cells. (**b**) Simulated trajectories of cell age and stochastic protein expression of two identical reporters on a branched tree. Line colour indicates reporter expression in the same cell. (**c**) Cell age and reporter expression of an isolated cell lineage. (**d**) Comparison of simulated distributions of lineages and population snapshots. Exemplary simulations of the reactions (29) assume *k*_0_ = 10, *k*_*m*_ = 1, *k*_*s*_ = 10 for each reporter and lognormal-distributed interdivision times with unit mean and standard deviation.

We model such data by investigating the dynamics of a dividing population of cell agents. The state of each cell is given by its age and the number of intracellular molecules, which are tracked from birth to division. After cell division, the two daughters have zero age and inherit different proportions of the mother’s molecules through stochastic partitioning of molecules. Cells divide at random times, so divisions occur asynchronously, which results in distributed cell ages and molecule numbers across the population. Figure 2a illustrates the resulting branching process whose final state represents a snapshot of the population.

Elowitz *et al*. identified the sources of cell-to-cell variation using snapshots of cells expressing green and red fluorescent reporters^1^. Reporters expressed at different levels in the cell appear either red or green, a signature of intrinsic noise. Cells with similar reporter levels light up in various shades of yellow, a signature of extrinsic noise. In our model (Fig. 2a), intrinsic variation results from stochasticity in biochemical reactions and partitioning of molecules at division whereas cell age and variability in division timing provide a source of extrinsic noise (Fig. 2b). An isolated cell tracked over time (Fig. 2c) displays different levels of variability than a typical cell in the population (Fig. 2d). We begin with deriving an analytical framework to compare these different distributions.

### Agent-based framework for stochastic biochemical kinetics in growing cell populations

To each cell we associate an age *τ* that counts the time since its last division and a set of biochemical species *X*_1_, *X*_2_, $\ldots$, ${X}_{{N}_{S}}$ with molecule numbers $x={({x}_{1},{x}_{2},\ldots ,{x}_{{N}_{S}})}^{T}$. These species interact via a network of *R* intracellular biochemical reactions of the type

$${\nu }_{1,r}^{-}\,{X}_{1}+\cdots +{\nu }_{{N}_{S},r}^{-}\,{X}_{{N}_{S}}\to {\nu }_{1,r}^{+}\,{X}_{1}+\cdots +{\nu }_{{N}_{S},r}^{+}\,{X}_{{N}_{S}},$$

where ${\nu }_{ir}^{\pm }$ are the stoichiometric coefficients and $r=1,\ldots ,R$. In the following, we outline the master equation that allows to analytically study these networks in an agent-based context.

#### Master equation for the agent-based population

The state of a cell population is described by the snapshot density *n*(*τ*, *x*, *t*) counting the number of cells at time *t* with age between *τ* and *τ* + d*τ* and molecule counts *x*. Accordingly, the total number of cells in the population is given by

1 $$N(t)={\int }_{0}^{\infty }\,{\rm{d}}\tau \,\sum _{x}\,n(\tau ,x,t),$$

where the summation is over all possible molecule number configurations.

We assume that cells divide with an age-dependent rate *γ*(*τ*), which can be written in terms of the interdivision time distribution *φ*(*τ*_*d*_) via

2 $$\gamma (\tau )=\frac{\phi (\tau )}{{\int }_{\tau }^{\infty }\,{\rm{d}}{\tau }_{d}\,\phi ({\tau }_{d})}\mathrm{.}$$

The snapshot density evolves due to age-progression of cells, cell divisions and the change in their molecular numbers in biochemical reactions (see SI Sec. S1 for a detailed derivation)

3a $$(\frac{{\rm{\partial }}}{{\rm{\partial }}t}+\frac{{\rm{\partial }}}{{\rm{\partial }}\tau }+\gamma (\tau ))n(\tau ,x,t)={\mathbb{Q}}n(\tau ,x,t).$$

Here, the change in the molecule numbers per cell is expressed by the transition matrix ${\mathbb{Q}}$ acting as

3b $${\mathbb{Q}}n(\tau ,x,t)=\sum _{r=1}^{R}\,[{w}_{r}(x-{\nu }_{r})n(\tau ,x-{\nu }_{r},t)-{w}_{r}(x)n(\tau ,x,t)],$$

where ${({\nu }_{r})}_{i}={\nu }_{ir}^{+}-{\nu }_{ir}^{-}$ is the stochiometric vector of the *r*^*th*^ reaction. Cell birth is described by the boundary condition

3c $$n\mathrm{(0},x,t)=2\,{\int }_{0}^{\infty }\,{\rm{d}}\tau \,\sum _{x^{\prime} }\,B(x|x^{\prime} )\gamma (\tau )n(\tau ,x^{\prime} ,t),$$

whereby the mother cell is replaced with two daughter cells of zero age and its molecules being partitioned between them. The division kernel *B*(*x*|*x*′) is the probability of partitioning the molecule numbers *x*′ to *x* of any daughter cell and is given by

4 $$B(x|x^{\prime} )=\frac{1}{2}{B}_{1}(x|x^{\prime} )+\frac{1}{2}{B}_{2}(x|x^{\prime} ),$$

where *B*_1_ and *B*_2_ are the marginal probabilities for the two daughter cells to inherit *x* of the *x*′ molecules in the mother cell (see SI Sec. 1). Importantly, since the total amount is conserved in the division, we have *B*_2_(*x*|*x*′) = *B*_1_(*x*′ − *x*|*x*′), which implies that, on average, cells inherit equal amounts of molecules *E*_*B*_[*x*|*x*′] = *x*′/2.

Since resolving the time-evolution of the snapshot density is a formidable task, we concentrate on the long-term evolution of Eq. (3), which describes the exponential growth phase or balanced growth condition. In this limit, the total number of cells grows exponentially *N*(*t*)~*e*^*λt*^ with rate *λ* and the fraction of cells with a certain cell age and molecule content is constant

5 $${\rm{\Pi }}(\tau ,x)=\mathop{\mathrm{lim}}\limits_{t\to \infty }\frac{n(\tau ,x,t)}{N(t)},$$

due to the balance between cell births, divisions and the biochemical reactions. In the following, we summarise how to describe this distribution analytically.

#### Age-distribution and population growth rate

The fraction of cells with the same age in a snapshot is given by the age-distribution, which follows^34^

6 $${\rm{\Pi }}(\tau )=\mathop{\mathrm{lim}}\limits_{t\to \infty }\,\sum _{x}\,\frac{n(\tau ,x,t)}{N(t)}=2\lambda {e}^{-\lambda \tau }\,{\int }_{\tau }^{\infty }\,{\rm{d}}\tau ^{\prime} \,\phi (\tau ^{\prime} \mathrm{).}$$

The distribution *φ* characterises the interdivision times

7 $$\phi ({\tau }_{d})=\gamma ({\tau }_{d}){e}^{-{\int }_{0}^{{\tau }_{d}}{\rm{d}}\tau ^{\prime} \gamma (\tau ^{\prime} )},$$

as also seen from Eq. (2). The age distribution, Eq. (6), depends on the population growth rate *λ* that is uniquely determined by the Euler-Lotka equation

8 $$1=2\,{\int }_{0}^{\infty }\,{\rm{d}}{\tau }_{d}\,{e}^{-\lambda \tau }\phi ({\tau }_{d}\mathrm{).}$$

The above equations constitute the fundamental age-structure of dividing cell populations, which has been verified in experiments^18,35,36^.

#### Distribution of molecules for cells of the same age

We consider the total number of cells with age *τ* and molecule count *x* divided by the number of cells at that age. This conditional probability quantifies the likelihood of observing *x* molecules in a cell of age *τ* and is given by

9 $${\rm{\Pi }}(x|\tau )=\mathop{\mathrm{lim}}\limits_{t\to \infty }\frac{n(\tau ,x,t)}{{\sum }_{x}\,n(\tau ,x,t)}=\frac{{\rm{\Pi }}(\tau ,x)}{{\rm{\Pi }}(\tau )}\mathrm{.}$$

It can be verified^34^ that Π(*x*|*τ*) obeys

10a $$\frac{\partial }{\partial \tau }{\rm{\Pi }}(x|\tau )={\mathbb{Q}}{\rm{\Pi }}(x|\tau ),$$

which is similar to the chemical master equation (with time replaced by cell age). An important difference though, is that it has to be solved subject to a boundary condition that accounts for the cell divisions

10b $${\rm{\Pi }}(x|\mathrm{0)}=\sum _{x^{\prime} }\,B(x|x^{\prime} )\,{\int }_{0}^{\infty }\,{\rm{d}}{\tau }_{d}\,{\rm{\Pi }}(x^{\prime} |{\tau }_{d})\rho ({\tau }_{d}\mathrm{).}$$

The condition states that the distribution at cell birth is the distribution at cell division after partitioning (cf. Fig. 1a). The distribution under the integral sign

11 $$\rho ({\tau }_{d})=2\phi ({\tau }_{d}){e}^{-\lambda {\tau }_{d}},$$

is the interdivision time distribution in the population^36,37^. The distribution describes the interdivision times of cells with completed cell divisions and depends explicitly on the population growth rate *λ*.

#### Comparison with the lineage framework

A lineage tracks a single cell of the population over successive cell divisions (cf. Fig. 1a). In the long-term, it approaches a stable distribution denoted by *π*(*τ*, *x*) = *π*(*τ*)*π*(*x*|*τ*). The molecule number distribution for cells of the same age is then given by *π*(*x*|*τ*) and satisfies

12 $$\begin{array}{rclrcl}\frac{\partial }{\partial \tau }\pi (x|\tau ) & = & {\mathbb{Q}}\pi (x|\tau ), & \pi (x\mathrm{|0)} & = & \sum _{x^{\prime} }\,B(x|x^{\prime} )\,{\int }_{0}^{\infty }\,{\rm{d}}{\tau }_{d}\,{\rm{\Pi }}(x^{\prime} |{\tau }_{d})\phi ({\tau }_{d}\mathrm{).}\end{array}$$

The age-distribution *π*(*τ*) is given by

13 $$\pi (\tau )=\frac{1}{{E}_{\phi }[\tau ]}\,{\int }_{\tau }^{\infty }\,{\rm{d}}\tau ^{\prime} \phi (\tau ^{\prime} \mathrm{).}$$

Analogous expressions to Eq. (12) have been given in^21,25,38,39^. Our approach accounts for cell cycle variability through the interdivision time distribution *φ*(*τ*_*d*_) explicitly. Comparing the above equations with Eqs (6) and (10), we see that the lineage distributions are obtained from the population distributions by substituting the interdivision time distribution *ρ*(*τ*_*d*_) for *φ*(*τ*_*d*_) and the age-distribution Π(*τ*) for *π*(*τ*). The intuition behind this result is that cells in the population and lineage follow the same reaction dynamics but experience different levels of cell cycle fluctuations. In particular, comparing Eq. (6) with (13) and Eq. (7) with (11), we observe that slow dividing cells are exponentially suppressed in the population. Thus the stochastic dynamics of biochemical reactions can be analysed within a common framework whether in populations or lineages. Analytical expressions for special cases of the different age and interdivision distributions are derived in SI Sec. S2.

### Moment equations for the agent-based model

Solving for the full distribution is infeasible in many practical situations. Summary statistics such as mean molecule numbers and their variances present convenient alternatives as they are more amenable to analysis and we will focus on these quantities in the following.

#### Exact moment equations for cells of the same age

In brief, the moment equations are obtained by multiplying Eq. (10a) by *x* or *xx*^*T*^ and summing over all possible states. The results for the first and second moments are

14a $$\frac{\partial {E}_{{\rm{\Pi }}}[x|\tau ]}{\partial \tau }=\sum _{r=1}^{R}\,{\nu }_{r}{E}_{{\rm{\Pi }}}[{w}_{r}(x)|\tau ],$$

14b $$\frac{\partial {E}_{{\rm{\Pi }}}[x{x}^{T}|\tau ]}{\partial \tau }=\sum _{r=1}^{R}({\nu }_{r}{E}_{{\rm{\Pi }}}[{x}^{T}{w}_{r}(x)|\tau ]+{E}_{{\rm{\Pi }}}[x{w}_{r}(x)|\tau ]{\nu }_{r}^{T})+\sum _{r=1}^{R}({\nu }_{r}{E}_{{\rm{\Pi }}}[{w}_{r}(x)|\tau ]{\nu }_{r}^{T}\mathrm{).}$$

Interestingly, these equations are the same moment equations that appear in the study of systems without age-dependence (with age being replaced by the observational time). The key difference is the boundary condition subject to which the moment equations have to be solved. These conditions follow from Eq. (10b) and the conservation of molecules in Eq. (4), which implies *E*_*B*_[*x*|*x*′] = *x*′/2. They read

14c $${E}_{{\rm{\Pi }}}[x\mathrm{|0]}=\frac{1}{2}{E}_{\rho }[{E}_{{\rm{\Pi }}}[x|{\tau }_{d}]],$$

14d $${E}_{{\rm{\Pi }}}[x{x}^{T}\mathrm{|0]}={E}_{\rho }[{E}_{{\rm{\Pi }}}({E}_{B}[x^{\prime} {x^{\prime} }^{T}|x]|{\tau }_{d}\mathrm{)].}$$

The first condition states that, on average, molecule numbers need to double over one cell cycle. The second condition relates the second moments to the partitioning of molecules described by the division kernel, Eq. (4).

#### Exact moment equations for cells of unknown age

When the cell age is not explicitly known, age-averaged moments have to be considered. For this purpose, we consider the derivative of a function *f*(*τ*) and average it with respect to the age distribution Π(*τ*). Using Eq. (6), we compute the identity

15 $${\int }_{0}^{\infty }\,{\rm{d}}\tau \,{\rm{\Pi }}(\tau )\frac{\partial f(\tau )}{\partial \tau }=\lambda {E}_{{\rm{\Pi }}}[f(\tau )]+\lambda {E}_{\rho }(f(\tau )-2f\mathrm{(0)),}$$

where we integrated by parts assuming ${\mathrm{lim}}_{\tau \to \infty }\,f(\tau ){\rm{\Pi }}(\tau )=0$ and performed the derivative. The first term describes the effect of dilution due to population growth, while the second term is due to cell division.

The equation for the first moment, *E*_Π_[*x*] = *E*_Π_[*E*_Π_[*x*|*τ*]], is obtained by setting *f*(*τ*) = *E*_Π_[*x*|*τ*] in (15), combing the result with Eq. (14a) and using the boundary condition (14c). The mean number of molecules in the population satisfies,

16 $$\lambda {E}_{{\rm{\Pi }}}[x]=\sum _{r=1}^{R}\,{\nu }_{r}{E}_{{\rm{\Pi }}}[{w}_{r}(x\mathrm{)].}$$

Similarly, using Eq. (15) with *f*(*τ*) = *E*_Π_[*xx*^*T*^|*τ*], Eqs (14b) and (14d), the equation for the second moments becomes

17 $$\begin{array}{l}\lambda {E}_{{\rm{\Pi }}}[x{x}^{T}]+\frac{\lambda }{2}{E}_{\rho }[{E}_{{\rm{\Pi }}}(x{x}^{T}|{\tau }_{d})]-4{E}_{{\rm{\Pi }}}({{\rm{Cov}}}_{B}[x^{\prime} |x]|\tau )]\\ \begin{array}{rcl} & = & \sum _{r=1}^{R}({\nu }_{r}{E}_{{\rm{\Pi }}}[{x}^{T}{w}_{r}(x)]+{E}_{{\rm{\Pi }}}[x{w}_{r}(x)]{\nu }_{r}^{T})+\sum _{r=1}^{R}\,{\nu }_{r}{E}_{{\rm{\Pi }}}[{w}_{r}(x)]{\nu }_{r}^{T},\end{array}\end{array}$$

where the left hand side depends explicitly on the division-time distribution *ρ*. These equations cannot generally be solved because the hierarchy of moments is not closed when the propensities depend non-linearly on the molecule numbers. Another complication is that they depend on the moments for cells of known age, which have to be solved simultaneously. The exception is the case of a constant age-independent division rate for which cell ages and interdivision times are exponentially distributed and divisions are memoryless (see SI Sec. S3 for further details). In the Results, we present a tractable approximation to these equations.

## Results

To circumvent the moment-closure problem, we employ the linear noise approximation to decompose the noise into intrinsic and extrinsic components. In brief, the approximation assumes Gaussian fluctuations and provides closed-form expressions for the mean molecule numbers *E*_Π_[*x*|*τ*] and the covariance matrix Cov_Π_[*x*|*τ*] = *E*_Π_[*xx*^*T*^|*τ*] − *E*_Π_[*x*|*τ*]*E*_Π_[*x*^*T*^|*τ*] (see^40,41^ for details), which leads to the moment equations

18a $$\frac{\partial }{\partial \tau }{E}_{{\rm{\Pi }}}[x|\tau ]=\sum _{r=1}^{R}\,{\nu }_{r}{w}_{r}({E}_{{\rm{\Pi }}}[x|\tau ]),$$

18b $$\frac{\partial }{\partial \tau }{{\rm{Cov}}}_{{\rm{\Pi }}}[x|\tau ]={\mathscr{J}}(\tau ){{\rm{Cov}}}_{{\rm{\Pi }}}[x|\tau ]+{{\rm{Cov}}}_{{\rm{\Pi }}}[x|\tau ]\,{{\mathscr{J}}}^{T}(\tau )+{\mathscr{D}}(\tau ),$$

where the Jacobian ${\mathscr{J}}$ and the diffusion matrix ${\mathscr{D}}$ are defined as

19 $${{\mathscr{J}}}_{ij}(\tau )=\sum _{r=1}^{R}\,{\nu }_{ir}\frac{\partial {w}_{r}({E}_{{\rm{\Pi }}}[x|\tau ])}{\partial {E}_{{\rm{\Pi }}}[{x}_{j}|\tau ]},\,{{\mathscr{D}}}_{ij}(\tau )=\sum _{r=1}^{R}\,{\nu }_{ir}{\nu }_{jr}{w}_{r}({E}_{{\rm{\Pi }}}[x|\tau \mathrm{]).}$$

These quantities depend on the cell age through the mean molecule numbers *E*_Π_[*x*|*τ*]. Comparison of Eqs (18) with (14) shows that the moment equations are exact whenever the propensities are linear in the molecule numbers. In all other cases, they have to be considered as an approximation valid in the limit of large molecule numbers.

Next, we cast the boundary condition (14d) in terms of the covariance matrix Cov_Π_[*x*|*τ*], which leads to

20a $${E}_{{\rm{\Pi }}}[x\mathrm{|0]}=\frac{1}{2}{E}_{\rho }[{E}_{{\rm{\Pi }}}(x|{\tau }_{d})],$$

20b $$\begin{array}{rcl}\mathop{\underbrace{{{\rm{Cov}}}_{{\rm{\Pi }}}[{x}_{i},{x}_{j}\mathrm{|0]}}}\limits_{{\rm{variability}}\,{\rm{between}}\,{\rm{daughters}}} & = & \mathop{\underbrace{\frac{1}{4}{E}_{\rho }[{{\rm{Cov}}}_{{\rm{\Pi }}}({x}_{i},{x}_{j}|\tau )]}}\limits_{{\rm{variability}}\,{\rm{between}}\,{\rm{mother}}\,{\rm{cells}}}\\  &  & +\,\mathop{\underbrace{{E}_{\rho }[{E}_{{\rm{\Pi }}}({{\rm{Cov}}}_{B}[{x}_{i},{x}_{j}|x^{\prime} ]|\tau )]}}\limits_{{\rm{partitioning}}\,{\rm{of}}\,{\rm{molecules}}}\\  &  & +\,\mathop{\underbrace{\frac{1}{4}{{\rm{Cov}}}_{\rho }[{E}_{{\rm{\Pi }}}({x}_{j}|\tau ),{E}_{{\rm{\Pi }}}({x}_{j}|\tau )]}}\limits_{{\rm{cell}}\,{\rm{cycle}}\,{\rm{variability}}}\mathrm{.}\end{array}$$

The first term is the contribution due to fluctuations in the number of molecules before division. The second term denotes the variation due to random partitioning of molecules at cell division, while the third term stems from differences in the molecule numbers due to different cell cycle lengths. Eq. (20b) does not constitute a noise decomposition itselves because the variability between mothers depends implicitly on the variability between daughters. Instead, the second and third terms constitute sources of intrinsic and extrinsic cell-to-cell variability as we will show in the following.

### Decomposing noise into intrinsic and extrinsic contributions

To investigate how the different sources of variations affect biochemical reaction dynamics, we consider the synthesis and degradation of mRNA molecules and translation into proteins

21 $$\varnothing \mathop{\longrightarrow }\limits^{{k}_{0}}{\rm{mRNA}}\mathop{\longrightarrow }\limits^{{k}_{m}}\varnothing ,\,{\rm{mRNA}}\mathop{\longrightarrow }\limits^{{k}_{s}}{\rm{mRNA}}+{\rm{Protein}}.$$

We do not account for protein degradation explicitly in this model since stable proteins are diluted in the population by cell division, the effect of which we will study in the following. For simplicity, we assume that mRNA degradation is faster than the population growth such that the reactions can be approximated by a single reaction synthesising proteins in stochastic bursts. At the same time, for the purpose of the noise decomposition, we consider an additional, identical copy of the same network in the cell

22 $$\varnothing \mathop{\longrightarrow }\limits^{{k}_{0}}{m}_{1}\times {{\rm{Protein}}}_{1},\,\varnothing \mathop{\longrightarrow }\limits^{{k}_{0}}{m}_{2}\times {{\rm{Protein}}}_{2}\mathrm{.}$$

The stochastic burst size of the first and second copy are denoted by *m*_1_ and *m*_2_, respectively, and both follow a geometric distribution with mean *b* = *k*_*s*_/*k*_*m*_ (see Ref.^42^ and SI Sec. S4 for details of the burst approximation).

#### Mean number of proteins

Since the two reporter proteins are expressed identically in the cell, their mean expression levels must be the same. Denoting the protein numbers of the two reporters by *x*_1_ and *x*_2_, we have *E*_Π_[*x*_1_|*τ*] = *E*_Π_[*x*_2_|*τ*]. The rate equation (18a) for the average number of proteins for a cell of given age *τ* becomes

$$\frac{\partial {E}_{{\rm{\Pi }}}[{x}_{1}|\tau ]}{\partial \tau }={k}_{0}b\mathrm{.}$$

The solution that respects the boundary condition (20a) is

23 $${E}_{{\rm{\Pi }}}[{x}_{1}|\tau ]={k}_{0}b(\tau +{E}_{\rho }[\tau \mathrm{]).}$$

The number of proteins inherited after cell division (*τ* = 0) is thus *k*_0_*bE*_*ρ*_[*τ*], which depends on the mean interdivision time *E*_*ρ*_[*τ*] in the population.

#### Noise decomposition for two-reporter systems

For identical two-reporter systems, the total variance can be decomposed into intrinsic and extrinsic components

24 $${\rm{\Sigma }}(\tau )={{\rm{\Sigma }}}_{{\rm{int}}}(\tau )+{{\rm{\Sigma }}}_{{\rm{ext}}}(\tau \mathrm{).}$$

The individual contributions can be quantified using the two reporters^1^

25 $${{\rm{\Sigma }}}_{{\rm{int}}}(\tau )=\frac{1}{2}{E}_{{\rm{\Pi }}}[({x}_{1}-{x}_{2}{)}^{2}|\tau ],\,{{\rm{\Sigma }}}_{{\rm{ext}}}(\tau )={{\rm{Cov}}}_{{\rm{\Pi }}}[{x}_{1},{x}_{2}|\tau \mathrm{].}$$

Since these components are measured in the same cell, these expressions also account for the correct history dependence^8,10,43^.

The variance of intrinsic and extrinsic fluctuations follows from using Eq. (25) in (18b) with ${\mathscr{J}}=0$ and ${\mathscr{D}}={k}_{0}{\sum }_{m=1}^{{\rm{\infty }}}\,{m}^{2}p(m)$, where *p*(*m*) is the geometric distribution (see SI Sec. S4 for details). Rearranging then leads to

$$\frac{\partial }{\partial \tau }{{\rm{\Sigma }}}_{{\rm{int}}}(\tau )=b\mathrm{(2}b+\mathrm{1)}{k}_{0},\,\frac{\partial }{\partial \tau }{{\rm{\Sigma }}}_{{\rm{ext}}}(\tau )=0.$$

Integrating these equations we find Σ_int_(*τ*) = Σ_int_(0) + *b*(2*b* + 1)*k*_0_*τ* and Σ_ext_(*τ*) = Σ_ext_(0). To match the boundary condition (20b), we assume that each molecule of the mother cell is being partitioned with equal probability between the two daughter cells. In this case, the division kernel in Eq. (4) is binomial with covariance ${{\rm{Cov}}}_{B}[{x}_{i},{x}_{j}|x^{\prime} ]={\delta }_{ij}{x^{\prime} }_{i}/4$. We then find that ${{\rm{\Sigma }}}_{{\rm{int}}}(0)=\frac{1}{3}b\mathrm{(2}b+\mathrm{3)}{k}_{0}{E}_{\rho }[\tau ]$ and ${{\rm{\Sigma }}}_{{\rm{ext}}}(0)=\frac{1}{3}{b}^{2}{k}_{0}^{2}{E}_{\rho }{[\tau ]}^{2}{{\rm{CV}}}_{\rho }^{2}$, and finally

26a $${{\rm{CV}}}_{{\rm{int}}}^{2}(\tau )=\frac{{{\rm{\Sigma }}}_{{\rm{int}}}(\tau )}{{E}_{{\rm{\Pi }}}{[{x}_{1}|\tau ]}^{2}}=\frac{1}{{E}_{{\rm{\Pi }}}[{x}_{1}|\tau ]}(1+\frac{2}{3}b[1+\frac{2\tau }{{E}_{\rho }[\tau ]+\tau }]),$$

26b $${{\rm{CV}}}_{{\rm{ext}}}^{2}(\tau )=\frac{{{\rm{\Sigma }}}_{{\rm{ext}}}(\tau )}{{E}_{{\rm{\Pi }}}{[{x}_{1}|\tau ]}^{2}}=\frac{{E}_{\rho }{[\tau ]}^{2}}{{({E}_{\rho }[\tau ]+\tau )}^{2}}\frac{{{\rm{CV}}}_{\rho }^{2}}{3}\mathrm{.}$$

The coefficients of variations quantify the size of fluctuations relative to the mean. The result confirms the intuition that intrinsic noise, Eq. (26a), decreases with the mean number of molecules. The extrinsic noise component, however, reflects the variations in cell cycle duration ${{\rm{CV}}}_{\rho }^{2}$ that are transmitted to the protein levels, which are independent of the mean.

#### Snapshots display higher intrinsic but lower extrinsic noise levels than lineages

Next, we compare the statistics of snapshots with the lineage of an isolated cell over time. As explained in the Methods, we obtain the lineage statistic by substituting the interdivision time distribution *ρ* for *φ* in Eqs (23), (26a) and (26b). Interestingly, the deviations between these two statistics is apparent even on the mean level. To see this, we notice that the mean number of molecules, Eq. (23), increases with the duration of the cell cycle. It is well known that the interdivision time is longer when averaged over single cells than for cells in the population^18^ *E*_*ρ*_[*τ*] ≤ *E*_*φ*_[*τ*]. An intuitive explanation of this fact is that fast dividing cells are over-represented in the population. It hence follows from Eq. (23) that the expected number of molecules is lower in populations compared to lineages, no matter what the interdivision time distribution is.

**Figure 3:**
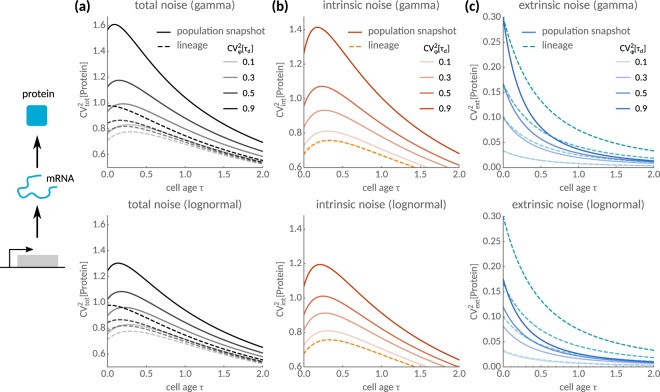
Intrinsic and extrinsic noise propagation over the cell cycle. (**a**) Total noise as a function of cell age *τ* with gamma (top) and log-normal-distributed (bottom) interdivision times. Population snapshot statistics (solid) are compared to lineages (dashed lines). (**b**) Intrinsic noise peaks as a function of cell age and increases with cell cycle fluctuations in populations but not in lineages. (**c**) Extrinsic noise is lower in the population than in lineages. Parameters are *k*_0_ = 1, *b* = 100 and interdivision time distributions assume unit mean.

In Fig. 3a, we compare the total noise ${{\rm{CV}}}_{{\rm{tot}}}^{2}(\tau )={{\rm{CV}}}_{{\rm{int}}}^{2}(\tau )+{{\rm{CV}}}_{{\rm{ext}}}^{2}(\tau )$ for gamma and log-normal distributed interdivision times. In both cases, we observe that the noise exhibits a maximum for low cell cycle variability. With increasing cell cycle variability, we find that the maximum flattens in the lineage but not in snapshot statistics. Albeit the two statistics are collected from different samples of the same population, snapshots are more noisy than lineages in both cases. To understand this noise propagation, we decompose the total noise into intrinsic and extrinsic components via Eqs (26a) and (26b). We observe that intrinsic noise in snapshots increases with cell cycle variability (Fig. 3b) while it is significantly lower in lineages and independent of these fluctuations, which is consistent with lower expression levels in snapshots.

Figure 3b reveals a non-monotonic dependence of the intrinsic noise component on cell age. To explain this phenomenon, we notice that intrinsic noise, Eq. (26a), increases with cell age due to an increase in the Fano factor (round brackets in Eq. (26a)). For older cells, however, intrinsic noise decreases with age as these cells express higher protein levels. Combining these findings explains the noise maximum at a well-defined cell age. By maximising Eq. (26a) over all possible cell ages, the age at which noise peaks is $\tau /{E}_{\rho }[{\tau }_{d}]=\frac{2b-3}{6b+3}$ whenever *b* > 3/2. This ratio only depends on the burst size *b* and approaches 1/3 of the mean interdivision time for large *b*. By contrast, we find that extrinsic noise is lower and decays slower over the cell cycle in snapshots than in lineages (Fig. 3c). We conclude that lineage statistics may significantly underestimate intrinsic heterogeneity but overestimate extrinsic noise in the population. In the next subsection, we extend this method to general stochastic reaction networks.

*General decomposition for cells of the same age*. Our aim is to generalise the decomposition to two-reporter systems involving an arbitrary network of biochemical reactions. As before, we assume that the two copies of our network with molecule numbers *x*_1_ and *x*_2_ do not interact and involve the same type reactions, and therefore the subnetworks have the same mean expression levels and covariances. The covariance matrix of the combined two-reporter system is

$${{\rm{Cov}}}_{{\rm{\Pi }}}[{x}_{1},{x}_{2}|\tau ]=(\begin{array}{ll}{{\rm{\Sigma }}}_{11}(\tau ) & {{\rm{\Sigma }}}_{12}(\tau )\\ {{\rm{\Sigma }}}_{12}(\tau ) & {{\rm{\Sigma }}}_{11}(\tau )\end{array}),$$

whose sub-matrices satisfy

27 $$\begin{array}{ll}\frac{\partial }{\partial \tau }{{\rm{\Sigma }}}_{11}(\tau )={\mathscr{J}}(\tau ){{\rm{\Sigma }}}_{11}+{{\rm{\Sigma }}}_{11}{{\mathscr{J}}}^{T}(\tau )+{\mathscr{D}}(\tau ), & \frac{\partial }{\partial \tau }{{\rm{\Sigma }}}_{12}(\tau )={\mathscr{J}}(\tau ){{\rm{\Sigma }}}_{12}+{{\rm{\Sigma }}}_{12}{{\mathscr{J}}}^{T}(\tau ),\end{array}$$

according to Eq. (18b).

The intrinsic and extrinsic noise components can be calculated as

$${{\rm{\Sigma }}}_{{\rm{int}}}(\tau )=\frac{1}{2}E[({x}_{1}-{x}_{2}{)}^{2}|\tau ]={{\rm{\Sigma }}}_{11}-{{\rm{\Sigma }}}_{12},\,{{\rm{\Sigma }}}_{{\rm{ext}}}(\tau )={\rm{Cov}}[{x}_{1},{x}_{2}|\tau ]={{\rm{\Sigma }}}_{12}\mathrm{.}$$

Since the overall covariance matrix obeys the linear equations (27), the two noise contributions evolve independently. In particular, the intrinsic and extrinsic covariance matrices satisfy

28a $$\frac{{\rm{d}}}{{\rm{d}}\tau }{{\rm{\Sigma }}}_{{\rm{int}}}(\tau )={\mathscr{J}}(\tau ){{\rm{\Sigma }}}_{{\rm{int}}}+{{\rm{\Sigma }}}_{{\rm{int}}}{{\mathscr{J}}}^{T}(\tau )+{\mathscr{D}}(\tau ),$$

28b $$\frac{{\rm{d}}}{{\rm{d}}\tau }{{\rm{\Sigma }}}_{{\rm{ext}}}(\tau )={\mathscr{J}}(\tau ){{\rm{\Sigma }}}_{{\rm{ext}}}+{{\rm{\Sigma }}}_{{\rm{ext}}}{{\mathscr{J}}}^{T}(\tau \mathrm{).}$$

Only the intrinsic component involves the biochemical noise from the intracellular reactions through the diffusion matrix ${\mathscr{D}}$.

For binomial partitioning, the covariance matrix is given by Cov_*B*_[*x*_1_, *x*_2_|*x*′] = diag(*x*′)/4, where diag(*x*′) is the diagonal matrix with entries *x*′ on the diagonal. This dependence allows us to split the boundary condition (20b) according to Eq. (24), which results in two independent conditions

28c $$4{{\rm{\Sigma }}}_{{\rm{int}}}(0)=\mathop{\underbrace{{E}_{\rho }[{{\rm{\Sigma }}}_{{\rm{int}}}({\tau }_{d})]}}\limits_{{\rm{intrinsic}}\,{\rm{noise}}\,{\rm{in}}\,{\rm{mother}}}+\mathop{\underbrace{{E}_{\rho }[{E}_{{\rm{\Pi }}}[{\rm{diag}}(x)|{\tau }_{d}]]}}\limits_{{\rm{partitioning}}\,{\rm{noise}}},$$

28d $$4{{\rm{\Sigma }}}_{{\rm{ext}}}\mathrm{(0)}=\mathop{\underbrace{{E}_{\rho }[{{\rm{\Sigma }}}_{{\rm{ext}}}({\tau }_{d})]}}\limits_{{\rm{extrinsic}}\,{\rm{noise}}\,{\rm{in}}\,{\rm{mother}}}+\mathop{\underbrace{{{\rm{Cov}}}_{\rho }[{E}_{{\rm{\Pi }}}[x|{\tau }_{d}],{E}_{{\rm{\Pi }}}[x|{\tau }_{d}]]}}\limits_{{\rm{cell}}\,{\rm{cycle}}\,{\rm{noise}}}\mathrm{.}$$

The partitioning noise features in Eq. (28c) as a source of intrinsic noise since molecules are partitioned independently between the daughter cells. The cell cycle noise represents a source of extrinsic noise in Eq. (28d) since the division timing of the mother cells affects both daughter cells in the same way. The noise decomposition is fully specified by the mean number of molecules for cells of the same age *E*_Π_[*x*|*τ*], the Jacobian ${\mathscr{J}}$ of the corresponding rate equations, the diffusion matrix ${\mathscr{D}}$ (see Eq. (19)) and the distribution of interdivision times in the population *ρ* (see Eq. (11)). Importantly, Eq. (28c) shows that partitioning is a noise source to intrinsic fluctuations, while Eq. (28d) shows that cell cycle variations contribute to extrinsic fluctuations. We conclude that conditioning on the cell cycle position is not enough to eliminate all extrinsic noise. Before we continue, we note that other types of partitioning, such as asymmetric cell division, can be incorporated into the framework by using a different form of Cov_*B*_[*x*_*i*_, *x*_*j*_|*x*′]^44^.

*General decomposition for cells of unknown age*. An obstacle for applying this decomposition in practice is that the cell age is often unknown, and this is especially true for population snapshots. For this reason, the mean of the molecule number has to be averaged over all possible cell ages

$${E}_{{\rm{\Pi }}}[x]={E}_{{\rm{\Pi }}}({E}_{{\rm{\Pi }}}[x|\tau ])={\int }_{0}^{\infty }\,{\rm{d}}\tau \,{E}_{{\rm{\Pi }}}[x|\tau ]{\rm{\Pi }}(\tau \mathrm{).}$$

Similarly, we use the law of total variance to decompose the snapshot-variance as

29a $$\bar{{\rm{\Sigma }}}=\mathop{\underbrace{{\bar{{\rm{\Sigma }}}}_{{\rm{i}}{\rm{n}}{\rm{t}}}}}\limits_{{\rm{i}}{\rm{n}}{\rm{t}}{\rm{r}}{\rm{i}}{\rm{n}}{\rm{s}}{\rm{i}}{\rm{c}}}+\mathop{\underbrace{{\bar{{\rm{\Sigma }}}}_{{\rm{c}}{\rm{c}}}+{\bar{{\rm{\Sigma }}}}_{{\rm{a}}{\rm{g}}{\rm{e}}}}}\limits_{{\rm{e}}{\rm{x}}{\rm{t}}{\rm{r}}{\rm{i}}{\rm{n}}{\rm{s}}{\rm{i}}{\rm{c}}},$$

with

29b $${\bar{{\rm{\Sigma }}}}_{{\rm{int}}}={E}_{{\rm{\Pi }}}({{\rm{\Sigma }}}_{{\rm{int}}}(\tau )),\,{\bar{{\rm{\Sigma }}}}_{{\rm{cc}}}={E}_{{\rm{\Pi }}}({{\rm{\Sigma }}}_{{\rm{ext}}}(\tau )),\,{\bar{{\rm{\Sigma }}}}_{{\rm{age}}}={{\rm{Cov}}}_{{\rm{\Pi }}}({E}_{{\rm{\Pi }}}[x|\tau ],{E}_{{\rm{\Pi }}}[x|\tau \mathrm{]).}$$

The first term in Eq. (29a) is the intrinsic variance measured across a population, the second term is the extrinsic variance transmitted from cell cycle variations, and the third term is another extrinsic component that comes from averaging over cells of different ages. The total extrinsic noise, which is measured in a two-reporter system, is the sum of the second and third term. The practical use of this noise decomposition is demonstrated in the following section.

### Practical computation of the noise decomposition and applications

Finally, we apply the noise decomposition to analyse snapshots with unknown cell ages. While the decomposition can be carried out exactly for linear reaction networks, we also outline an efficient numerical method for complex nonlinear networks and demonstrate its efficacy for a protein that regulates its own expression.

#### Decomposition for linear reaction networks in cells of unknown age

Linear reaction networks have propensities functions that are linear functions of the molecule numbers. This dependence implies *E*_Π_[*w*_*r*_(*x*)] = *w*_*r*_(*E*_Π_[*x*]) and allows averaging the statistics exactly over all cell ages. Thus from Eq. (16), we obtain the rate equations

30 $$\lambda {E}_{{\rm{\Pi }}}[x]=\sum _{r=1}^{R}\,{\nu }_{r}{w}_{r}({E}_{{\rm{\Pi }}}[x]),$$

which coincide with the steady state of the traditional deterministic rate equations including an effective dilution term proportional to the population growth rate *λ*.

Averaging Eq. (28a) over all ages and accounting for the boundary terms using Eq. (15), the intrinsic variance becomes

31a $$\lambda {\bar{{\rm{\Sigma }}}}_{{\rm{int}}}={\mathscr{J}}\,{\bar{{\rm{\Sigma }}}}_{{\rm{int}}}+{\bar{{\rm{\Sigma }}}}_{{\rm{int}}}{{\mathscr{J}}}^{T}+{E}_{{\rm{\Pi }}}[{\mathscr{D}}(\tau )]+\lambda (2{{\rm{\Sigma }}}_{{\rm{int}}}\mathrm{(0)}-{E}_{\rho }[{{\rm{\Sigma }}}_{{\rm{int}}}({\tau }_{d})])\mathrm{.}$$

Here, the Jacobian ${\mathscr{J}}$ is independent of cell age because the propensities are linear. Similarly, averaging Eq. (28b) the extrinsic variance transmitted from cell cycle fluctuations is obtained as

31b $$\lambda {\bar{{\rm{\Sigma }}}}_{{\rm{cc}}}={\mathscr{J}}\,{\bar{{\rm{\Sigma }}}}_{{\rm{cc}}}+{\bar{{\rm{\Sigma }}}}_{{\rm{cc}}}{{\mathscr{J}}}^{T}+\lambda (2{{\rm{\Sigma }}}_{{\rm{ext}}}\mathrm{(0)}-{E}_{\rho }[{{\rm{\Sigma }}}_{{\rm{ext}}}({\tau }_{d})])\mathrm{.}$$

An equation for ${\bar{{\rm{\Sigma }}}}_{{\rm{age}}}$ can also be derived (SI Sec. S5), which reads

31c $$\lambda {\bar{{\rm{\Sigma }}}}_{{\rm{a}}{\rm{g}}{\rm{e}}}={\mathscr{J}}\,{\bar{{\rm{\Sigma }}}}_{{\rm{a}}{\rm{g}}{\rm{e}}}+{\bar{{\rm{\Sigma }}}}_{{\rm{a}}{\rm{g}}{\rm{e}}}{{\mathscr{J}}}^{T}+\lambda ({E}_{{\rm{\Pi }}}[x]{E}_{{\rm{\Pi }}}[{x}^{T}]-2{E}_{{\rm{\Pi }}}[x|0]{E}_{{\rm{\Pi }}}[{x}^{T}|0]-\,{{\rm{C}}{\rm{o}}{\rm{v}}}_{\rho }({E}_{{\rm{\Pi }}}[x|{\tau }_{d}],{E}_{{\rm{\Pi }}}[x|{\tau }_{d}])).$$

This decomposition describes the variability of linear reaction networks across snapshots exactly.

#### Application to stochastic reporter expression

We return to the two-reporter system (22) and apply the decomposition developed in the previous section. From Eq. (30), we find that the mean molecule number is given by

32 $$E[p]=\{\begin{array}{ll}{k}_{0}b/\lambda  & {\rm{snapshot}},\\ b{k}_{0}({E}_{\phi }[{\tau }_{d}]+{E}_{\pi }[\tau ]) & {\rm{lineage}}\mathrm{.}\end{array}$$

**Figure 4:**
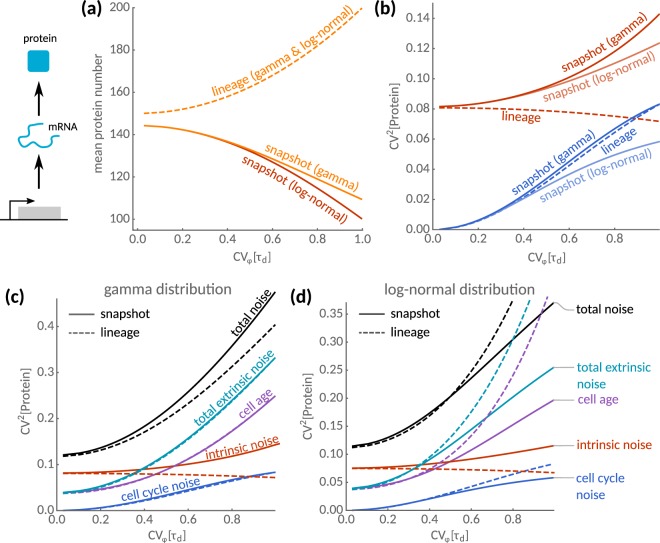
Statistics of population snapshots and isolated lineages for cells of unknown age. (**a**) Mean protein number as a function of the cell cycle variations CV_*φ*_[*τ*_*d*_] in lineages (dashed) and snapshots (solid lines). For lineages, the mean protein number increases with cell cycle variability and is independent of the interdivision time distribution. In snapshots, the mean decreases with cell cycle variability with a rate that depends on higher moments of the distribution. The predictions for gamma- and log-normal distributed interdivision times are shown. (**b**) Sensitivity of intrinsic and extrinsic noise sources to cell cycle fluctuations. Intrinsic noise (red lines) increases in lineages but decreases in snapshots consistent with the dependence of the respective means shown in (**a**). The transmitted cell cycle noise (blue lines) shows a similar dependence on cell-cycle variability in lineages and snapshots for the gamma-distribution, but is lower in snapshots for the log-normal distribution. (**c**) Total noise (black lines) broken down into individual noise components for the gamma-distribution. Transmitted cell cycle noise and the uncertainty due to distributed cell ages (purple lines) contribute to the total extrinsic noise (teal). (**d**) The corresponding decomposition for the log-normal distribution. Parameters are *k*_0_ = 10 and *b* = 10 and *E*_*φ*_[*τ*_*d*_] = 1.

Note that the lineage mean follows from integrating Eq. (23) with *φ* instead of *ρ* against the lineage age-distribution (13). Both averages depend implicitly on the cell cycle variability through the average age *E*_*π*_[*τ*] or the population growth rate *λ*, respectively (see SI Sec. 2). Interestingly, the population mean is limited by the growth rate but the lineage mean depends both on age and interdivision time. In Fig. 4a, we show that, due to this dependence, molecule numbers increase with cell cycle variability in lineages while they decrease when using snapshot statistics under the same conditions. These quantities thus exhibit opposite sensitivities to cell cycle variability.

Next, we explore the noise properties of the reporter system using the decomposition (31). We find that the contributions of intrinsic noise are

$${{\rm{CV}}}_{{\rm{int}}}^{2}=\{\begin{array}{ll}\frac{1}{{E}_{{\rm{\Pi }}}[p]}(1+\frac{2}{3}b(1+2\lambda {E}_{{\rm{\Pi }}}[\tau ])) & {\rm{snapshot}},\\ \frac{1}{{E}_{\pi }[p]}(1+2b-\frac{4b{E}_{\phi }[{\tau }_{d}]}{3({E}_{\phi }[{\tau }_{d}]+{E}_{\pi }[\tau ])}) & {\rm{lineage}},\end{array}$$

which are inversely proportional to the mean number of proteins. The contribution of extrinsic noise due to stochasticity in cell cycle duration is

$${{\rm{CV}}}_{{\rm{cc}}}^{2}=\{\begin{array}{ll}\frac{1}{3}{({E}_{\rho }[{\tau }_{d}]\lambda )}^{2}{{\rm{CV}}}_{\rho }^{2}[{\tau }_{d}] & {\rm{snapshot}},\\ \frac{1}{3}\frac{{E}_{\phi }{[{\tau }_{d}]}^{2}}{{({E}_{\phi }[{\tau }_{d}]+{E}_{\pi }[\tau ])}^{2}}{{\rm{CV}}}_{\phi }^{2}[{\tau }_{d}] & {\rm{lineage}},\end{array}$$

and the one due to the unknown cell age is

$${{\rm{CV}}}_{{\rm{age}}}^{2}=\{\begin{array}{ll}{(\lambda {E}_{{\rm{\Pi }}}[\tau ])}^{2}{{\rm{CV}}}_{{\rm{\Pi }}}^{2}[\tau ] & {\rm{snapshot}},\\ \frac{{E}_{\pi }{[\tau ]}^{2}}{{({E}_{\phi }[{\tau }_{d}]+{E}_{\pi }[\tau ])}^{2}}{{\rm{CV}}}_{\pi }^{2}[\tau ] & {\rm{lineage}}\mathrm{.}\end{array}$$

The noise decomposition crucially depends on the population growth rate *λ*, while in lineages it depends on the corresponding average cell age *E*_*π*_[*τ*]. More specifically, ${{\rm{CV}}}_{{\rm{cc}}}^{2}$ and ${{\rm{CV}}}_{{\rm{age}}}^{2}$ depend on the variations in the age and interdivision time distributions, which are generally different in lineages and population (see SI Sec. S2 for details). We illustrate this dependence using the analytical decompositions for two different interdivision-time distributions with the same mean and variance.

For both the gamma and the log-normal distribution, intrinsic noise (red lines, Fig. 4b) exhibits opposite sensitivities on cell cycle variability comparing lineage (dashed) and snapshot statistics (solid). This observation is explained by smaller mean expression levels in snapshots (cf. Fig. 4a) because intrinsic noise is expected to scale inversely with the mean molecule number. For the gamma distribution, the extrinsic noise transmitted from cell cycle variations (blue lines, Fig. 4b) is (almost) identical for these measures. For the log-normal distribution, however, extrinsic noise in the lineage is smaller than in the snapshot. Interestingly, we find that the total noise is higher in snapshots than in lineages for the gamma distribution (black lines, Fig. 4c), while this not true for the log-normal distribution and large cell cycle variability (black lines, Fig. 4d).

In developed network models, the extrinsic components will also depend on the biochemical properties of the network. We demonstrate this analytically in SI Sec. S5 when the protein is also subject to degradation, which reveals more intricate noise patterns. A straight-forward approach for the noise decomposition in nonlinear biochemical networks is given in the following section.

#### Decomposition for nonlinear reaction networks

For nonlinear reaction networks, it is generally difficult to carry out the noise decomposition analytically. This is because the statistics of known and unknown cell age are intricately coupled and can be solved simultaneously only in simple cases. An efficient and generally applicable algorithm to compute the noise decomposition numerically is the following:

1. Calculate the population growth rate using Eq. (8).
2. Solve for the statistics of cells of the same age, Eqs (28a) and (28b), and use the shooting method to match the boundary conditions (28c) and (28d).
3. Obtain the noise decomposition (29a) irrespectively of cell age by performing the averages in Eq. (29b).

Step 1 can be efficiently computed using numerical root-finding methods. The shooting method in Step 2 consists of an iterative procedure by which the mean molecule number, intrinsic variance and extrinsic variance are obtained using an ODE solver with an initial guess on their values at cell birth, ${E}_{{\rm{\Pi }}}$[*x*|0], Σ_int_(0) and Σ_ext_(0), and refining the result using standard root-finding methods until the boundary conditions (28c) and (28d) are matched. Step 3 is easily carried out alongside the numerical integration of Step 2. The procedure typically evaluates the noise decomposition in seconds on a desktop computer and may therefore be adequate for large parameter sweeps required for statistical inference.

#### Suppressing intrinsic or extrinsic noise through feedback mechanisms

Over 40% of known transcription factors in *E*. *coli* regulate their own expression^45^. We here investigate the sensitivity of negative autoregulatory feedback to cell cycle fluctuations. We consider transcription and degradation of mRNA molecules from which proteins are synthesised

33 $$\varnothing \mathop{\longrightarrow }\limits^{h(p)}{\rm{mRNA}}\mathop{\longrightarrow }\limits^{{k}_{1}}\varnothing ,\,{\rm{mRNA}}\mathop{\longrightarrow }\limits^{{k}_{2}}{\rm{mRNA}}+{\rm{Protein}}\mathrm{.}$$

The effect of negative feedback is modelled via a Hill-function for the transcription rate $h(p)=\frac{{k}_{0}}{1+{(p/K)}^{4}}$, an approximation that implicitly assumes fast promoter-binding^46^. The transcription rate decreases with the protein number *p* depending on the effective dissociation constant *K*.

**Figure 5:**
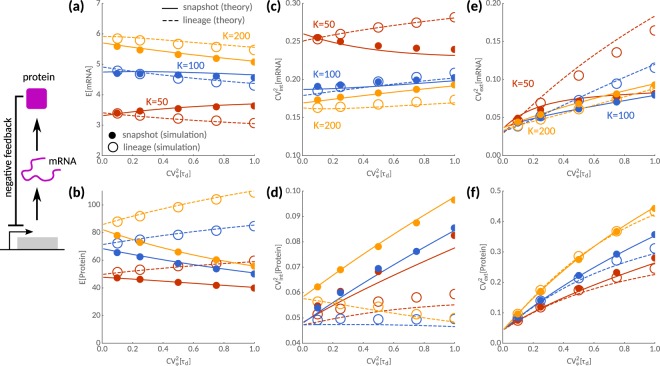
Noise decomposition of a negative feedback circuit. Sensitivities to cell cycle noise ${{\rm{CV}}}_{\phi }^{2}[{\tau }_{d}]$ of mean, intrinsic and extrinsic noise contributions are shown for weak (yellow, *K* = 200), moderate (blue, *K* = 100) and strong feedback (red, *K* = 50). Predictions by the linear noise approximation (solid lines) are in good qualitative agreement with stochastic simulations (dots). (**a**) In lineages, the mean mRNA number always decreases with cell cycle variability while this is not true in snapshots for moderate to high feedback. (**b**) In contrast, protein levels always increase in lineages but decrease in snapshots. (**c**) The corresponding intrinsic noise profiles of mRNAs typically increase with cell cycle noise except in snapshots with strong feedback. (**d**) Intrinsic noise of proteins always increases with cell cycle noise in snapshots but not in lineages. (**e**,**f**) Total extrinsic noise increases with cell cycle variability for mRNAs and proteins. However, strong feedback may significantly reduce extrinsic noise in snapshots compared to lineages. Deviations between the approximation (lines) and the simulations (dots) are most pronounced for strong feedback. Parameters are *k*_0_ = 10, *k*_*m*_ = 1, *k*_*s*_ = 10 and log-normal distributed interdivision times with unit-mean.

In Fig. 5a we show that, in lineages, mRNA levels decrease with cell cycle variability for various feedback strength (the inverse of the dissociation constant, 1/*K*). Mean mRNA numbers in the snapshot statistic either decrease (weak, moderate feedback) or increase with cell cycle variability (strong feedback) depending on the feedback strength. In contrast, protein levels increase with cell cycle variability in lineages but decrease when using snapshot statistics for various feedback strengths (Fig. 5b). In agreement with this trend, we find that intrinsic noise in protein expression increases with cell cycle variability in snapshots while the opposite behaviour is observed in lineages for weak to moderate feedback (Fig. 5d). Strikingly, due to the negative feedback regulation, the sensitivity of intrinsic noise of mRNAs and proteins follows an inverse relationship (Fig. 5c), but the differences between lineage and snapshot statistics are most pronounced for weak feedback. In contrast to the intrinsic noise properties of the circuit, the total extrinsic noise of the circuit always increases with cell cycle variability, both in lineages and snapshots (Fig. 5d,e). In all cases, our approximations are in good agreement with exact stochastic simulations of two non-interacting reporter networks (Fig. 5 dots) carried out using the First-Division Algorithm^34^.

Finally, we use the noise decomposition to understand how heterogeneity can be controlled in natural and synthetic circuits by sweeping the feedback strength parameter (1/*K*). Negative feedback is widely known to reduce noise but often requires fine-tuned parameters^47,48^. How this translates to individual functional noise components, such as intrinsic and extrinsic noise, has only been explored in response to fluctuations in parameters and shared components^7,16,49^ but not in the context of cell division dynamics. Here, we are specifically interested in the sensitivity of lineage and population snapshot statistics to cell cycle noise.

**Figure 6:**
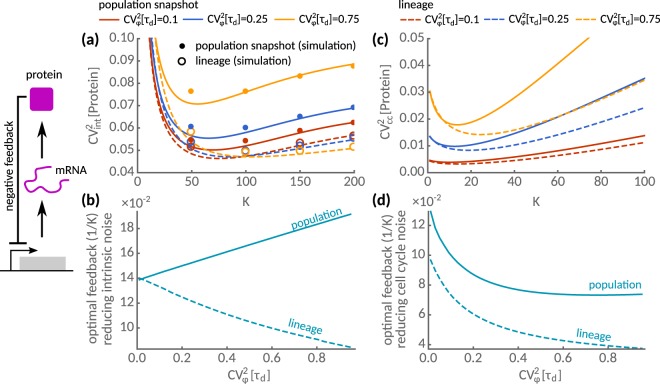
Feedback strategies for noise suppression in lineages and populations. Intrinsic and extrinsic noise statistics of negative autoregulatory feedback circuit are shown as a function of *K*, the inverse feedback strength, for three different levels of cell cycle noise ${{\rm{CV}}}_{\phi }^{2}[{\tau }_{d}]=0.1$ (red), 0.25 (blue) and 0.75 (yellow). (**a**) Intrinsic noise exhibits a minimum as a function of the repression strength both in lineage (dashed) and in snapshot statistics (solid lines). The predictions obtained using the linear noise approximation (lines) are in good agreement with exact stochastic simulations using the First-Division Algorithm^34^ (dots for population, open circles for lineages). (**b**) Optimal feedback strength (1/*K*) to minimise intrinsic noise is shown. The feedback strength increases with cell cycle noise in lineages but decreases in population snapshots. (**c**) The transmitted cell cycle noise shows a minimum in dependence of the repression strength both in lineage (dashed) and in snapshot statistics (solid lines). (**d**) The optimal feedback strength to minimise transmitted cell cycle noise decreases with interdivision time noise both in lineages and to a lesser extent in the population. Parameters as in Fig. 5.

In Fig. 6a, we show that negative feedback can efficiently suppress intrinsic noise as the feedback strength is varied. Intriguingly, comparing the minimum noise levels in lineages and snapshots, vastly different values of the dissociation constants achieve noise suppression in these measures. To study this dependency in more detail, we compute the optimal feedback strength that minimises intrinsic noise as shown in Fig. 6b. Intriguingly, the optimal values exhibit opposite sensitivities to the cell cycle variability in lineages than in the population snapshots. To efficiently suppress intrinsic noise in a lineage, we must decrease the feedback strength in response to an increase in cell cycle variability. To compensate for intrinsic variability across the population, however, the feedback strength must increase by almost a two-fold of what would be required in the lineage.

In other situations, it may be advantageous to reduce the extrinsic instead of the intrinsic noise component. Tuning the dissociation constant (*K*) can reduce the transmitted cell cycle noise ${{\rm{CV}}}_{cc}^{2}$ (Fig. 6c). Comparing the optimal feedback strength (1/*K*) as a function of the cell cycle noise CV_*φ*_[*τ*_*d*_] (Fig. 6d), we observe that it increases with cell cycle variability in both lineages and snapshots. Vastly different feedback strengths, however, achieve either intrinsic or extrinsic noise suppression (cf. Fig. 6b,d). In population snapshots, we need to increase the feedback strength to decrease noise, while in lineages we need to decrease it. These findings highlight that a single feedback loop is not sufficient to suppress several functional noise components in lineages and population snapshots simultaneously.

## Discussion

We present an analytical framework to quantify stochastic biochemical reactions in an exponentially growing cell population. This theory allows us to characterise and systematically decompose noise in general biochemical reaction networks into intrinsic and extrinsic components. We found that a typical cell in the population expresses lower protein levels per cell than an isolated cell tracked over successive cell divisions. The intuition behind this effect is that fast-growing cells are overrepresented in populations, where cells compete for growth. Since these cells accumulate less proteins over the cell cycle, their overall expression decreases. As a consequence, we observed higher levels of intrinsic noise but, for the examples studied, the extrinsic noise component was significantly reduced. These effects are most pronounced in the presence of interdivision time variability as it is expected in natural populations. Importantly, this highlights that one needs to account for cell cycle fluctuations when modelling intrinsic or extrinsic noise.

Previous studies^38,39^ focussed on the effect of age-structure but mostly neglected cell cycle variations. If these variations are taken into account the statistics of lineages and population snapshots are not equivalent even when the cell cycle position is known. This implies that measuring cells within narrow stages of the cell cycle as achieved through gating^9,31^, for instance, does not eliminate all extrinsic noise due to cell cycle fluctuations. In reality, cells are affected by more than one type of extrinsic noise source as reaction rates may fluctuate over time and between cells^7,8,14^. These effects should be added to the transmitted extrinsic noise. We anticipate, however, that it will be difficult in practice to distinguish these fluctuations from the variations induced by cell cycle variability.

While this study focused on the effects of age-structure on biochemical dynamics, several simulation studies suggest that cell size also coordinates gene expression^50,51^. Incorporating additional physiological details such as cell size into our framework could thus provide insights to the statistics of intracellular concentrations^52–54^ and extrinsic noise transduced from cell size control and growth rate fluctuations^55^. A different limitation of this study is that it is based on the linear noise approximation, which albeit being exact for linear reaction networks, represents an approximation assuming large molecule numbers. Its estimates can be inaccurate for nonlinear reaction networks involving low numbers of molecules. An improvement to this approximation could employ higher order terms in the system size expansion^46,56^, or close the hierarchy of moments using moment closure approximations^57^.

Statistics obtained from snapshots are often used to say something about a cell’s history. By grouping cells of similar ages, as in ergodic rate analysis^58^, one can in principle reconstruct time-course information. We demonstrated that such a procedure produces different results to the lineage statistic (see Fig. 3). Instead, the variability across the population is equivalent to choosing an arbitrary cell from the final population and tracing it backwards in time^34^. This equivalence provides a sample-path interpretation of snapshot data, but it is worth pointing out that it does not apply when cell ages are unidentified. In this case, understanding the relationship between single cell fluctuations and population heterogeneity requires an agent-based framework as the one presented here.

We showed that gene expression noise is coupled to the population growth rate and cell cycle variability. With increasing cell cycle variability, fast-growing cells become increasingly overrepresented in population snapshots because these cells divide faster. In contrast, slow-growing cells become increasingly frequent in isolated lineages because cells are tracked over time. Since faster growing cells produce less molecules, mean molecule levels decrease with cell cycle variability in populations but increase in lineages. Taking into account this dependency implies that cellular heterogeneity displays opposite sensitivities to cell cycle variability across populations and lineages. Thus, reducing intrinsic noise in lineages over time thus comes at the cost of increasing population heterogeneity.

Cells may exploit negative feedback circuits to control this variability. Our findings imply, however, that cells cannot minimise noise of lineages and populations simultaneously (Fig. 6). Since minimising noise over time increases population heterogeneity, cells may use this strategy to diversify in response to stress^59^. Conversely, tuning population homogeneity sacrifices lineage-optimality, which could confer advantages when gene expression couples to global physiological factors such as cell size, growth rate or cell division^3,60^. Identifying the relevant noise components and cellular objectives will likely depend on the environmental or experimental conditions^59,61^. These dependencies reveal a fundamental trade-off for the evolution of natural circuits and the design of synthetic circuits in living cells.

While we assumed that exponential population growth, our results hold identically when the population size is kept constant as long as the population size is sufficiently large (100–1000 cells are sufficient in our experience). This includes chemostats where cells are randomly removed. In summary, we presented an agent-based framework that reveals typical features of snapshot data using noise decompositions. The theory shows that noise statistics vary with the experimental setup to grow the cells, whether it is a mother machine or a chemostat. The framework is widely applicable and as such it also applies to large gene regulatory, signalling or metabolic networks. We, therefore, envision that the proposed moment-based approach could prove especially useful for parameter inference from snapshots of living cells^62^.

## Electronic supplementary material

Supplementary Information

## Data Availability

All data is available from the corresponding author upon request.
