# Supplementary material for: Intrinsic and extrinsic noise of gene expression in lineage trees
Source: Sci Rep. 2019 Jan 24;9:474. doi: 10.1038/s41598-018-35927-x (PMC6345792; doi:10.1038/s41598-018-35927-x)
Supplement: Supplementary file 1 — Supplementary Information [file 41598_2018_35927_MOESM1_ESM.pdf]

Supplementary Information: Intrinsic and extrinsic noise of gene expression in lineage trees

Philipp Thomas[1,*]

[1]Department of Mathematics, Imperial College London, London SW7 2AZ, UK
[*]p.thomas@imperial.ac.uk

NOTE

Equations of the main text are referenced as MEq.

Supplementary Information 1: Master equation for the agent-based framework

We derive the master equation governing the agent-based framework of the main text. We consider the sample paths $\{(\tau_i(t), x_i(t))_{i=1,..,N(t)}\}_{t\geq 0}$ of $N(t)$ cells, where $\tau_i(t)$ is the age of the ith cell and $x_i(t) = (x_{1,i}(t), ..., x_{N_S,i}(t))^T$ is its vector of molecule numbers. We denote by $\mathbb{P}$ the probability measure on which the overall population branching process is defined. First, we consider the probability $\mathbb{P}(s)$ to observe a particular snapshot $s(\tau, x, t)$ given by the empirical density function

$$s(\tau,x,t) = \sum_{i=1}^{N(t)} \delta(\tau - \tau_i)\delta_{x,x_i}, \tag{S1}$$

which counts the number of cells with age τ and molecule numbers x at time t. Implicitly, we assume a fixed initial condition $s(\tau, x, 0)$. Writing short $s(t + \Delta t) = s(x, \tau + \Delta t, t + \Delta t)$ and assuming small Δt, this probability evolves according to

$$\begin{aligned}\mathbb{P}(s(t+\Delta t)) =& \mathbb{P}(s(t)) \\ &+ \Delta t \left[\sum_{r=1}^{R} \int \mathrm{d}\Delta s_r \, \mathbb{P}(s(t) - \Delta s_r)\Omega_{\Delta s_r}(s(t) - \Delta s_r) - \mathbb{P}(s(t)) \sum_{r=1}^{R} \int \mathrm{d}\Delta s_r \, \Omega_{\Delta s_r}(s(t)) \right] \\ &+ \Delta t \left[\int \mathrm{d}\Delta s_d \, \mathbb{P}(s(t) - \Delta s_d)\Omega_{\Delta s_d}(s(t) - \Delta s_d) - \mathbb{P}(s(t)) \int \mathrm{d}\Delta s_d \, \Omega_{\Delta s_d}(s(t)) \right] + O(\Delta t^2),\end{aligned} \tag{S2}$$

where Δs_r in the second line denotes a change in the snapshot due to one of the R reactions and Δs_d in the third line denotes a change due to cell division. $\Omega_{\Delta s_r}$ and $\Omega_{\Delta s_d}$ denote the corresponding transition rates.

We will now evaluate the *snapshot density* given by the expectation

$$n(\tau,x,t) = E_{\mathbb{P}}[s(t)]. \tag{S3}$$

Intuitively, the snapshot density characterises the statistics obtained from integrating different realizations of the snapshot $s(t)$ and averaging out the fluctuations in the number of cells. Equivalently, it also characterises a single snapshot with a large number of cells $N(t)$ as obtained in the asymptotic limit $t \to \infty$ (meaning $N(t) \to \infty$) considered in the main text. It follows from Eq. (S2) that, in the limit $\Delta t \to 0$, the snapshot density obeys

$$\frac{\mathrm{d}n(\tau,x,t)}{\mathrm{d}t} = \frac{\mathrm{d}E_{\mathbb{P}}[s(t)]}{\mathrm{d}t} = \sum_{r=1}^{R} E_{\mathbb{P}} \left[\int \mathrm{d}\Delta s_r \, \Delta s_r \Omega_{\Delta s_r}(s(t)) \right] + E_{\mathbb{P}} \left[\int \mathrm{d}\Delta s_d \, \Delta s_d \Omega_{\Delta s_d}(s(t)) \right], \tag{S4}$$

with $n(0) = E_{\mathbb{P}}[s(0)]$. The equation states that the rate of change of the average snapshot is given by the expected changes due cell division and the reactions of single snapshots averaged over all possible snapshots.

Transitions due to reactions

Since a reaction occurs in a cell with propensity $w_r(x')$ and reactions occur independently in each cell, the propensity for a reaction to occur in cells of age τ' with molecule numbers x' is

$$\Omega_{\Delta s_r}(\tau', x', t) = w_r(x')s(\tau', x', t). \tag{S5}$$

If such a reaction fires, a cell with x' molecules is replaced with a cell with $x'+\nu_r$ molecules and the snapshot changes according to

$$\Delta s_r(\tau,x|\tau',x') = -\delta(\tau-\tau')\delta_{x,x'} + \delta(\tau-\tau')\delta_{x,x'+\nu_r}, \tag{S6}$$

where ν_r is the stoichiometric vector of the reaction. The expected change in a snapshot then follows from

$$\begin{aligned}\int \mathrm{d}\Delta s_r \Delta s_r \Omega_{\Delta s_r}(s(t)) &= \sum_{x'}\int_0^\infty \mathrm{d}\tau'\,\Delta s_r(\tau,x|\tau',x')\Omega_r(\tau',x',t) = \sum_{x'}\int_0^\infty \mathrm{d}\tau'\,\Delta s_r(\tau,x|\tau',x')w_r(x')s(\tau',x',t)\\ &= -w_r(x)s(\tau,x,t) + w_r(x-\nu_r)s(\tau,x-\nu_r,t),\end{aligned} \tag{S7}$$

such that

$$E_{\mathbb{P}}\left[\int \mathrm{d}\Delta s_r\,\Delta s_r \Omega_{\Delta s_r}(s(t))\right] = -w_r(x)n(\tau,x,t) + w_r(x-\nu_r)n(\tau,x-\nu_r,t). \tag{S8}$$

Transitions due to cell divisions

In our model, cell divisions occur with age-dependent propensity $\gamma(\tau')$ and independently for each cell. It then follows that the division propensity for a cell of age τ' with x' molecules is

$$\Omega_{\Delta s_d}(\tau',x',t) = \gamma(\tau')s(\tau',x',t). \tag{S9}$$

In the division, we replace a cell of age τ' and x' molecules with two cells of age 0 and x_1 and x_2 molecules, respectively. The snapshot then changes as

$$\Delta s_{x_1,x_2}(\tau,x|\tau',x') = -\delta(\tau-\tau')\delta_{x,x'} + \delta(\tau)\delta_{x,x_1} + \delta(\tau)\delta_{x,x_2}. \tag{S10}$$

Since the molecules are partitioned according to the joint probability $B(x_1,x_2|x)$, the expected change is

$$\begin{aligned}\int \mathrm{d}\Delta s_d \Delta s_d \Omega_{\Delta s_d}(s(t)) =& \sum_{x_1}\sum_{x_2}\sum_{x'}\int_0^\infty \mathrm{d}\tau'\,B(x_1,x_2|x')\Delta s_{x_1,x_2}(\tau,x|\tau',x')w(\tau',x',t)\\ =& -\gamma(\tau)s(\tau,x,t)\\ &+\delta(\tau)\int_0^\infty \mathrm{d}\tau'\sum_{x'}\sum_{x_2}B(x,x_2|x')\gamma(\tau')s(\tau',x',t) + \delta(\tau)\int_0^\infty \mathrm{d}\tau'\sum_{x'}\sum_{x_1}B(x_1,x|x')\gamma(\tau')s(\tau',x',t)\\ =& -\gamma(\tau)s(\tau,x,t) + 2\delta(\tau)\int_0^\infty \mathrm{d}\tau'\sum_{x'}B(x|x')\gamma(\tau')s(\tau',x',t),\end{aligned} \tag{S11}$$

where the division kernel $B(x|x')$ corresponds to the average of the two daughter cells

$$B(x|x') = \frac{1}{2}\sum_{x_1}B(x_1,x|x') + \frac{1}{2}\sum_{x_2}B(x,x_2|x'). \tag{S12}$$

It then follows that

$$E_{\mathbb{P}}\left[\int \mathrm{d}\Delta s_d\,\Delta s_d \Omega_{\Delta s_d}(s(t))\right] = -\gamma(\tau)n(\tau,x,t) + 2\delta(\tau)\int_0^\infty \mathrm{d}\tau'\sum_{x'}B(x|x')\gamma(\tau')n(\tau',x',t). \tag{S13}$$

Master equation for the snapshot density

We now combine the contributions of cell divisions and reactions to write a master equation for the snapshot density $n(\tau,x,t) = E_{\mathbb{P}}[s(t)]$. Using the total derivative, we have

$$\frac{\mathrm{d}n(\tau,x,t)}{\mathrm{d}t} = \frac{\partial n(\tau,x,t)}{\partial t} + \frac{\partial n(\tau,x,t)}{\partial \tau}, \tag{S14}$$

where the derivative with respect to τ corresponds to the age progression of cells. Using this relation in Eq. (S2) together with Eqs. (S8) and (S13), we find

$$\begin{aligned}\frac{\partial n(\tau,x,t)}{\partial t} + \frac{\partial n(\tau,x,t)}{\partial \tau} + \gamma(\tau)n(\tau,x,t) =& \sum_{r=1}^{R}\left(w_r(x-\nu_r)n(\tau,x-\nu_r,t) - w_r(x)n(\tau,x,t)\right)\\ &+2\delta(\tau)\int_0^\infty \mathrm{d}\tau'\sum_{x'}B(x|x')\gamma(\tau')n(\tau',x',t)\end{aligned} \tag{S15}$$

The terms in the last line can be absorbed into a boundary condition. To see this, we integrate Eq. (S15) over a small window of ages from $-\varepsilon$ to ε and take the limit $\varepsilon \to 0$. The result is

$$\lim_{\varepsilon\to 0}\int_{-\varepsilon}^{\varepsilon} \mathrm{d}\tau \frac{\partial n(\tau,x,t)}{\partial \tau} = n(0,x,t) = 2\int_0^\infty \mathrm{d}\tau' \sum_{x'} B(x|x')\gamma(\tau')n(\tau',x',t), \tag{S16}$$

since $n(x,-\varepsilon,t) = 0$. The solution to Eq. (S15) is thus equivalent to MEqs. (3) of the main text. An equivalent derivation can be found in[1].

Supplementary Information 2: Statistics of interdivision times and age distributions

Here, we study the statistics of division times and age-distributions in lineages and populations. In the following, we make use of the Laplace transform of the interdivision time distribution $\varphi(\tau_d)$, which is

$$\hat{\varphi}(s) = \int_0^\infty \mathrm{d}\tau_d \, e^{-s\tau_d}\varphi(\tau_d), \tag{S17}$$

and assume that $\varphi(\tau_d)$ is a known distribution or can be calculated from the division rate via MEq. (7).

Moments of interdivision times in a population

The distribution of interdivision times in the population is $\rho(\tau_d) = 2e^{-\lambda\tau_d}\varphi(\tau_d)$, MEq. (11), and its Laplace transform is

$$\hat{\rho}(s) = 2\hat{\varphi}(\lambda + s). \tag{S18}$$

Note that $\hat{\varphi}(\lambda) = 1/2$ due to characteristic equation (8). Its n-th moment is obtained by differentiating the Laplace transform

$$E_\rho[\tau_d^n] = 2\left(-\frac{\partial}{\partial\lambda}\right)^n \hat{\varphi}(\lambda), \tag{S19}$$

from which we compute mean, variance

$$E_\rho[\tau_d] = -2\hat{\varphi}'(\lambda), \;\; \mathrm{Var}_\rho[\tau_d] = 2\hat{\varphi}''(\lambda) - 4\hat{\varphi}'(\lambda)^2, \tag{S20}$$

and the coefficient of variation

$$\mathrm{CV}^2_\rho[\tau_d] = \frac{\mathrm{Var}_\rho[\tau_d]}{E_\rho[\tau_d]^2} = \frac{\hat{\varphi}''(\lambda)}{2\hat{\varphi}'(\lambda)^2} - 1. \tag{S21}$$

Age-distribution in lineages

The age-distribution gives the frequency of observed cell ages. To compute its moments, we use the Laplace transform of MEq. (13), which reads

$$\hat{\pi}(s) = \frac{1-\hat{\varphi}(s)}{sE_\varphi[\tau_d]}. \tag{S22}$$

By differentiating the above expression repeatedly at $s = 0$, we find

$$E_\pi[\tau] = \frac{E_\varphi[\tau^2]}{2E_\varphi[\tau]}, \;\; \mathrm{Var}_\pi[\tau] = \frac{E_\varphi[\tau^3]}{3E_\varphi[\tau]} \tag{S23}$$

and

$$\mathrm{CV}^2_\pi[\tau] = \frac{4E_\varphi[\tau]E_\varphi[\tau^3]}{3E_\varphi[\tau^2]}, \tag{S24}$$

which characterise the age-statistics.

Age-distribution in populations

Similarly, we consider the age-distribution in a population snapshot, MEq. (6), whose Laplace transform evaluates to

$$\hat{\Pi}(s) = E_\Pi[e^{-s\tau}] = 2\lambda \int_0^\infty \mathrm{d}\tau\, \Phi(\tau) e^{-(s+\lambda)\tau} = \frac{2\lambda}{s+\lambda}\left(1-\hat{\varphi}(s+\lambda)\right). \tag{S25}$$

Repeated differentiation at $s = 0$, gives

$$E_\Pi[\tau] = \frac{1}{\lambda} + 2\hat{\varphi}'(\lambda), \;\; \mathrm{Var}_\Pi[\tau] = \frac{1}{\lambda^2} - 2\hat{\varphi}''(\lambda) - 4\hat{\varphi}'(\lambda)^2. \tag{S26}$$

These expressions can be evaluated if the Laplace transform of the distribution is known. If this is not the case, as for the log-normal distribution (see Fig. 3 and 4 of the main text), we can evaluate these expressions numerically. In the following, we provide explicit expressions for the population growth rate, the age- and interdivision-time distributions in the case of the gamma distribution.

Gamma distribution: Computing age and interdivision time distributions and moments

The density function of the gamma distribution is

$$\varphi(\tau_d) = \frac{e^{-\frac{\tau_d}{c\mu}}\left(\frac{\tau_d}{c\mu}\right)^{1/c}}{\Gamma\left(\frac{1}{c}\right)\tau_d}, \tag{S27}$$

where the constants $\mu = E_\varphi[\tau_d] =$ and $c = \mathrm{CV}^2_\varphi[\tau_d]$ parameterise mean and squared coefficient of variation and Γ is the gamma function.

Growth rate. The Laplace transform is

$$\hat{\varphi}(s) = (c\mu s + 1)^{-1/c}. \tag{S28}$$

Recasting the Euler-Lotka equation in the form $\hat{\varphi}(\lambda) = 1/2$ and solving for λ, we obtain

$$\lambda = \frac{2^c - 1}{c\mu}. \tag{S29}$$

Interdivision times. The interdivision time distribution in the population then also follows a gamma distribution

$$\rho(\tau_d) = 2e^{-\lambda\tau_d}\varphi(\tau_d) = \frac{2e^{-\frac{2^c\tau_d}{c\mu}}\left(\frac{\tau_d}{c\mu}\right)^{1/c}}{\Gamma\left(\frac{1}{c}\right)\tau_d}, \tag{S30}$$

but it has a shorter mean division time

$$E_\rho[\tau_d] = 2^{-c}\mu, \;\; \mathrm{CV}^2_\rho[\tau_d] = c. \tag{S31}$$

Age distributions. Finally, we compute the age-distributions. The result for the lineage is

$$\pi(\tau) = \frac{1}{\mu}\frac{\Gamma\left(\frac{1}{c}, \frac{\tau}{c\mu}\right)}{\Gamma\left(\frac{1}{c}\right)}, \tag{S32}$$

where $\Gamma(\cdot,\cdot)$ is the upper incomplete gamma function. Its statistics are

$$E_\pi[\tau] = \frac{\mu}{2}(1+c), \;\; \mathrm{CV}^2_\pi[\tau] = \frac{5c+1}{3c+3}. \tag{S33}$$

Similarly, the age-distribution in the population becomes

$$\Pi(\tau) = 2\lambda e^{-\lambda\tau}\frac{\Gamma\left(\frac{1}{c}, \frac{\tau}{c\mu}\right)}{\Gamma\left(\frac{1}{c}\right)} \tag{S34}$$

with statistics

$$E_\Pi[\tau] = \mu\left(\frac{c}{2^c-1} - \frac{1}{2^c}\right),$$

$$\mathrm{CV}^2_\Pi[\tau] = \frac{2c}{c+2^{-c}-1} - \frac{(2^c-1)^2(c+1)}{(2^c(c-1)+1)^2} - 1. \tag{S35}$$

Interestingly, it follows that $E_\Pi[\tau] < E_\pi[\tau]$, but $\mathrm{CV}^2_\Pi[\tau] > \mathrm{CV}^2_\pi[\tau]$ for $c < 1$ and $\mathrm{CV}^2_\Pi[\tau] < \mathrm{CV}^2_\pi[\tau]$ for $c > 1$.

Supplementary Information 3: Detailed discussion of the moment-closure conditions

While the moment equations derived in the Methods are exact, the equations for cells of the same age are only closed when $w_r(x)$ depends at most linearly on the molecule numbers x and the covariance of the partitioning kernel $\mathrm{Cov}_B[x|x']$ depends at most quadratically on the number molecules in the mother cell x'. This holds, for instance, for biochemical composed solely from unimolecular reactions and independent binomial partitioning. Similarly, it holds true for the mean of cells with unknown age, but not generally for their corresponding variances. Specifically, the covariance for cells of unknown age also depends on the moments for cells of known age and thus they must explicitly depend on the division time distribution.

There are now two scenarios in which the variances are independent of the division time distribution. The first case is when the age-distribution coincides with the division time distribution $\Pi(\tau) = \rho(\tau)$, which follows only when the division rate γ is constant and independent of age, i.e. the division times are exponentially distributed. The second case assumes a particular division kernel $B(x|x')$ that satisfies $\mathrm{Cov}_B[x|x'] = \frac{1}{4}x'x'^T$, which follows when all molecules are inherited by only one of the daughter cells. In all other cases, which seem most relevant in practice, the moment equations for unknown cell age involve the moments for cells of known age. Thus, for general nonlinear reaction networks, they involve two hierarchies of moments that cannot be easily closed. A simple and generally applicable approximation is given in the main text that circumvents this problem using the linear noise approximation.

Supplementary Information 4: Analytical noise decomposition for gene expression with degradation

We consider a simple network in which a protein P is translated in stochastic bursts of size m and is subsequently degraded

$$\varnothing \xrightarrow{k_0} m \times P, \quad P \xrightarrow{k_1} \varnothing. \tag{S36}$$

Because the burst size is a random variable with distribution $q(m)$, we can recast the synthesis reaction into a series of reactions with reaction rates $k_0 q(m)$,

$$\varnothing \xrightarrow{k_0 q(1)} 1 \times P, \quad \varnothing \xrightarrow{k_0 q(2)} 2 \times P, \quad \varnothing \xrightarrow{k_0 q(3)} 3 \times P, \quad \ldots, \tag{S37}$$

$$P \xrightarrow{k_1} \varnothing. \tag{S38}$$

For the two-stage model of gene expression, q is the geometric distribution.

Mean protein number of the same age. The equation for the mean number of molecules is then

$$\frac{\partial E_\Pi[x|\tau]}{\partial \tau} = k_0\left(\sum_{m=1}^{\infty} m q(m)\right) - k_1 E_\Pi[x|\tau] = b k_0 - k_1 E_\Pi[x|\tau] \tag{S39}$$

with solution

$$E_\Pi[x|\tau] = E_\Pi[x|0] e^{-k_1\tau} + \frac{b k_0}{k_1}\left(1 - e^{-k_1\tau}\right). \tag{S40}$$

Substituting the solution into the boundary condition $E_\rho(E_\Pi[x|\tau]) = 2E_\Pi[x|0]$ and solving for $E_\Pi[x|0]$ yields the final result

$$E_\Pi[x|\tau] = \frac{b k_0}{k_1}\left(1 - \frac{e^{-k_1\tau}}{2 - \hat{\rho}(k_1)}\right), \tag{S41}$$

where $\hat{\rho}$ is the Laplace transform of the division time distribution

$$\hat{\rho}(s) = \int_0^\infty \mathrm{d}\tau_d\, e^{-s\tau_d} \rho(\tau_d). \tag{S42}$$

We will see in the following that also the noise statistics depends crucially on this function.

Protein fluctuations for cells of the same age. To compute the protein fluctuations, we observe from (S41), that the Jacobian is

$$\mathscr{J} = -k_1. \tag{S43}$$

The diffusion matrix then follows from MEq. (19) then follows

$$\mathscr{D} = k_0 \sum_{m=1}^{\infty} m^2 q(m) + k_1 E_\Pi[x|\tau] = k_0 b(2b+1) + k_1 E_\Pi[x|\tau], \tag{S44}$$

assuming geometric bursts. The variance of intrinsic and extrinsic fluctuations obeys MEqs. (18b)

$$\frac{\partial}{\partial \tau}\Sigma_{\text{int}}(\tau) = -2k_1 \Sigma_{\text{int}}(\tau) + k_0 b(2b+1) + k_1 E_\Pi[x|\tau], \tag{S45}$$

$$\frac{\partial}{\partial \tau}\Sigma_{\text{ext}}(\tau) = -2k_1 \Sigma_{\text{ext}}(\tau). \tag{S46}$$

The solutions are

$$\begin{aligned}\Sigma_{\text{int}}(\tau) &= \frac{bk_0 e^{-k_1 t}}{k_1\left(\hat{\rho}(k_1)-2\right)} + \frac{b(b+1)k_0}{k_1} \\ &\quad + e^{-2k_1 t}\left(\frac{bk_0\left(-(b+1)\hat{\rho}(k_1)+2b+1\right)}{k_1(\hat{\rho}(k_1)-2)} + \Sigma_{\text{int}}(0)\right)\end{aligned} \tag{S47}$$

$$\Sigma_{\text{ext}}(\tau) = \Sigma_{\text{ext}}(0)e^{-2k_1 t}, \tag{S48}$$

where $\Sigma_{\text{int}}(0)$ and $\Sigma_{\text{ext}}(0)$ are the intrinsic and extrinsic variation at cell division, which have to be determined from the boundary conditions. According to MEq. (28c) and (28d), the boundary conditions are

$$\begin{aligned}4\Sigma_{\text{int}}(0) &= E_\rho[\Sigma_{\text{int}}(\tau_d)] + E_\rho[E_\Pi[x|\tau_d]], \\ 4\Sigma_{\text{ext}}(0) &= E_\rho[\Sigma_{\text{ext}}(\tau_d)] + \text{Cov}_\rho[E_\Pi[x|\tau_d], E_\Pi[x|\tau_d]].\end{aligned} \tag{S49}$$

To compute these values we notice that the variances at cell division follow from averaging Eqs. (S47) over the division time distribution ρ, which results in

$$\begin{aligned}E_\rho[\Sigma_{\text{int}}(\tau_d)] &= \frac{bk_0\hat{\rho}(k_1)}{k_1\left(\hat{\rho}(k_1)-2\right)} + \frac{b(b+1)k_0}{k_1} + \hat{\rho}(2k_1)\left(\frac{bk_0\left(-(b+1)\hat{\rho}(k_1)+2b+1\right)}{k_1(\hat{\rho}(k_1)-2)} + \Sigma_{\text{int}}(0)\right) \\ E_\rho[\Sigma_{\text{ext}}(\tau_d)] &= \Sigma_{\text{ext}}(0)\hat{\rho}(2k_1).\end{aligned} \tag{S50}$$

Further, we evaluate

$$E_\rho(E_\Pi[x|\tau_d]) = 2E_\Pi[x|0] = 2\frac{bk_0}{k_1}\left(\frac{1-\hat{\rho}(k_1)}{2-\hat{\rho}(k_1)}\right), \tag{S51}$$

and

$$\begin{aligned}\text{Cov}_\rho[E_\Pi[x|\tau_d], E_\Pi[x|\tau_d]] &= E_\rho\left(E_\Pi[x|\tau] - E_\rho(E_\Pi[x|\tau])\right)^2 \\ &= \left(\frac{bk_0}{k_1(2-\hat{\rho}(k_1))}\right)^2 E_\rho\left(\hat{\rho}(k_1) - e^{-k_1\tau}\right)^2 = \left(\frac{bk_0}{k_1}\right)^2 \frac{\left(\hat{\rho}(2k_1)-\hat{\rho}^2(k_1)\right)}{(2-\hat{\rho}(k_1))^2}.\end{aligned} \tag{S52}$$

Substituting Eqs. (S50), (S51) and (S52) into (S49), solving for $\Sigma_{\text{int}}(0)$ and $\Sigma_{\text{ext}}(0)$ and using the result in Eqs. (S47), we finally arrive at

$$\begin{aligned}\Sigma_{\text{int}}(\tau) &= \frac{bk_0}{k_1}\left((b+1) - \frac{3be^{-2k_1 t}}{4-\hat{\rho}(2k_1)} - \frac{e^{-k_1 t}}{2-\hat{\rho}(k_1)}\right), \\ \Sigma_{\text{ext}}(\tau) &= \frac{b^2k_0^2}{k_1^2}\frac{\left(\hat{\rho}(2k_1)-\hat{\rho}^2(k_1)\right)e^{-2k_1 t}}{\left(\hat{\rho}(k_1)-2\right)^2\left(4-\hat{\rho}(2k_1)\right)},\end{aligned} \tag{S53}$$

which determines the progression of intrinsic and extrinsic fluctuations over the cell cycle.

Protein statistics for cells of unknown age. The mean protein number is given by

$$E_\Pi[x] = \frac{bk_0}{k_1+\lambda}. \tag{S54}$$

Thus the mean number is determined from the balance between the rates of translation, degradation and dilution due to cell divisions. From MEqs. (31) we compute

$$\bar{\Sigma}_{\text{int}} = \frac{b(b+1)k_0}{k_1} - \frac{b\lambda k_0}{\lambda k_1 + k_1^2} + \frac{3b^2\lambda k_0\left(\hat{\rho}\left(2k_1\right)-2\right)}{k_1\left(4-\hat{\rho}\left(2k_1\right)\right)\left(\lambda+2k_1\right)}, \tag{S55}$$

$$\bar{\Sigma}_{\text{ext}} = \frac{b^2\lambda k_0^2\left(\hat{\rho}\left(2k_1\right)-\hat{\rho}^2\left(k_1\right)\right)\left(2-\hat{\rho}\left(2k_1\right)\right)}{k_1^2\left(2-\hat{\rho}\left(k_1\right)\right)^2\left(4-\hat{\rho}\left(2k_1\right)\right)\left(\lambda+2k_1\right)}, \tag{S56}$$

$$\bar{\Sigma}_{\text{age}} = \frac{b^2\lambda k_0^2\left(2-\hat{\rho}\left(2k_1\right)\right)}{k_1^2\left(\hat{\rho}\left(k_1\right)-2\right)^2\left(\lambda+2k_1\right)} - \frac{b^2\lambda^2 k_0^2}{k_1^2\left(\lambda+k_1\right)^2}. \tag{S57}$$

Finally, we compute $\text{CV}_y^2 = \bar{\Sigma}_y / E_\Pi[x]^2$ to arrive at

$$\text{CV}_{\text{int}}^2 = \frac{1}{E_\Pi[x]}\left(1+b\left(1+\frac{b\lambda}{k_1}\right) - \frac{3b\lambda}{k_1}\frac{\left(2-\hat{\rho}\left(2k_1\right)\right)}{\left(4-\hat{\rho}\left(2k_1\right)\right)}\frac{\left(\lambda+k_1\right)}{\left(\lambda+2k_1\right)}\right), \tag{S58}$$

$$\text{CV}_{\text{cc}}^2 = \frac{\lambda\left(\hat{\rho}\left(2k_1\right)-\hat{\rho}\left(k_1\right)^2\right)\left(\hat{\rho}\left(2k_1\right)-2\right)\left(\lambda+k_1\right)^2}{k_1^2\left(\hat{\rho}\left(k_1\right)-2\right)^2\left(\hat{\rho}\left(2k_1\right)-4\right)\left(\lambda+2k_1\right)}, \tag{S59}$$

$$\text{CV}_{\text{age}}^2 = \frac{\lambda\left(2-\hat{\rho}\left(2k_1\right)\right)\left(\lambda+k_1\right)^2}{k_1^2\left(\hat{\rho}\left(k_1\right)-2\right)^2\left(\lambda+2k_1\right)} - \frac{\lambda^2}{k_1^2}, \tag{S60}$$

which denote the intrinsic noise, the transmitted noise from cell cycle fluctuations and the uncertainty due to the unknown cell age. It is evident that these expressions are more involved than for the case without degradation because they depend on the Laplace transform $\hat{\rho}$ of the division time distribution. Interestingly, the last two components, which together represent the extrinsic noise, also depend on the degradation rate k_1 meaning that the total extrinsic noise is constant only when the mean concentration is varied through the either transcription rate or burst size.

Supplementary Information 5: An explicit formula for the uncertainty due to unknown cell age

We here verify MEq. (31c) of the main text for the case of linear reaction networks. To this end we define $\varepsilon(\tau) = E_\Pi[x|\tau] - E_\Pi[x]$ such that $\bar{\Sigma}_{\text{age}} = \text{Cov}_\Pi[E[x|\tau]] = E_\Pi[\varepsilon\varepsilon^T]$ and use MEq. (18a) to write

$$\frac{\partial}{\partial\tau}\varepsilon(\tau) = \nu w(E[x|\tau]) = c + \mathscr{J}(\varepsilon(\tau) + E_\Pi[x]). \tag{S61}$$

We used the fact that, for linear reaction networks, the propensities are linear in the number of molecules, i.e., $\nu w(E[x|\tau]) = c + \mathscr{J} E[x|\tau]$ where c is a constant vector. Making use of Eq. (S61) we then compute

$$\begin{aligned} E_\Pi\left[\frac{\partial}{\partial\tau}\varepsilon(\tau)\varepsilon^T(\tau)\right] =& E_\Pi\left[\varepsilon(\tau)\frac{\partial}{\partial\tau}\varepsilon^T(\tau)\right] + E_\Pi\left[\varepsilon^T(\tau)\frac{\partial}{\partial\tau}\varepsilon(\tau)\right] \\ =& \mathscr{J}\bar{\Sigma}_{\text{age}} + \bar{\Sigma}_{\text{age}}\mathscr{J}^T. \end{aligned}$$

On the other hand, using MEq. (15) of the main text, it follows that

$$\begin{aligned} E_\Pi\left[\frac{\partial}{\partial\tau}\varepsilon(\tau)\varepsilon^T(\tau)\right] =& \lambda\left(\bar{\Sigma}_{\text{age}} + E_\rho[\varepsilon(\tau)\varepsilon^T(\tau)] - 2\varepsilon(0)\varepsilon^T(0)\right) \\ =& \lambda\left(\bar{\Sigma}_{\text{age}} + \text{Cov}_\rho[E[x|\tau]] - E_\Pi[x]E_\Pi[x^T] + 2E_\rho[x|0]E_\rho[x^T|0]\right). \end{aligned}$$

Combining the last two equation gives result (31c) of the main text.

References

1. Thomas, P. Making sense of snapshot data: ergodic principle for clonal cell populations. *J Royal Soc Interface* **14**, 20170467 (2017). DOI 10.1098/rsif.2017.0467.
